# Mechanisms of Resistance to MEK Inhibitors (RAS–MAPK Pathway) in Malignant Peripheral Nerve Sheath Tumors and Their Precursor Lesions (Plexiform Neurofibromas) Associated with Neurofibromatosis Type 1

**DOI:** 10.3390/cancers18182950

**Published:** 2026-09-11

**Authors:** Sergey I. Sologov, Diana Sologova, Denis Dubinin, George Anikin, Nana Bekhorashvili, Maria Rayisyan, Elena Krylova, Milada Yarkova, Ekaterina M. Grigorevskikh, Elena Smolyarchuk, Susanna Sologova

**Affiliations:** 1 Sklifosovsky Institute of Clinical Medicine, Sechenov First Moscow State Medical University (Sechenov University), Moscow 119991, Russia; sologov_s_i@student.sechenov.ru (S.I.S.); dubinin_d_d1@student.sechenov.ru (D.D.); anikin_g_s@staff.sechenov.ru (G.A.); 2 Department of Oral Surgery, Institute of Dentistry, Sechenov First Moscow State Medical University (Sechenov University), Moscow 119991, Russia; sologova_d_i@staff.sechenov.ru; 3 Department of Regulatory Relations in the Field of Circulation of Medicines and Medical Devices, Sechenov First Moscow State Medical University (Sechenov University), Moscow 119991, Russia; bekhorashvili_n@staff.sechenov.ru (N.B.); raysyan_m_g@staff.sechenov.ru (M.R.); 4 Department of Pharmacology, A.P. Nelyubin Institute of Pharmacy of Sechenov First Moscow State Medical University (Sechenov University), Moscow 119991, Russia; krylova_e_v_3@staff.sechenov.ru (E.K.); yarkova_m_a@staff.sechenov.ru (M.Y.); grigorevskikh_e_m@staff.sechenov.ru (E.M.G.); smolyarchuk_e_a@staff.sechenov.ru (E.S.)

**Keywords:** neurofibromatosis type 1, plexiform neurofibroma, malignant peripheral nerve sheath tumor, MEK inhibitor, selumetinib, mirdametinib, drug resistance, RAS–MAPK signaling, adaptive resistance, targeted therapy

## Abstract

Background/Objectives: MEK inhibitors (selumetinib, mirdametinib) are the only approved class of targeted therapy for neurofibromatosis type 1 (NF1)-associated plexiform neurofibroma (PN), producing partial responses in a substantial proportion of patients (up to 63.6% in adults; objective response rate 19.7% versus 5.4% with placebo in the KOMET trial). Responses, however, are rarely complete or durable, and in malignant peripheral nerve sheath tumor (MPNST) single-agent MEK inhibition is clinically ineffective. Mechanisms of escape from MEK inhibition in these tumors remain poorly characterized and are reported in the literature as isolated primary studies without an integrative analysis. The aim of this review was to systematize both the established molecular mechanisms of resistance to MEK inhibitors in PN and MPNST and the biologically plausible candidate mechanisms extrapolated from other RAS-driven malignancies. Methods: This is a narrative review. A structured search was performed in PubMed, PubMed Central, NCBI Bookshelf, and Scopus, supplemented by clinical practice guidelines and regulatory documents; it covered publications up to 31 May 2026 and was updated in August 2026. Ninety-six sources are cited, and their composition by publication type is reported; record counts at the intermediate screening steps were not maintained, and no PRISMA flow diagram is presented. Results: Mechanisms were classified within a convergent framework into six categories: reactivation of MAPK signaling within the cascade; parallel (bypass) reactivation through receptor tyrosine kinases and adjacent inputs; epigenetic and transcriptional rewiring; cell survival programs; the tumor microenvironment and immune evasion; and intratumoural heterogeneity. Each mechanism was then graded along two independent axes—the strength of evidence that it confers resistance in PN or MPNST (E1–E3) and its therapeutic tractability (T1–T3)—and annotated with the entity and model constituting its evidence source. Conclusions: Resistance to MEK inhibition in PN and MPNST is convergent rather than mechanism-unique. The principal limitation of the field is the near-absence of clinical resistance data from patients progressing on MEK inhibitors; prospective molecular monitoring, rational combination trials, and mechanism-stratifying biomarkers are the priorities.

## Simple Summary

Neurofibromatosis type 1 is the most common inherited tumor predisposition syndrome. Between a third and a half of affected people are born with plexiform neurofibromas—benign but disfiguring nerve tumors that arise during development, usually become clinically apparent during childhood as they enlarge, and in a minority of cases transform into an aggressive cancer called malignant peripheral nerve sheath tumor. Two drugs, selumetinib and mirdametinib, are the only approved targeted treatments for these tumors. They shrink plexiform neurofibromas in a substantial proportion of patients, but the tumor almost never disappears completely, and in the malignant form the drugs barely work at all. The biological reasons why tumors escape these drugs are scattered across many individual studies and have never been brought together. In this review, we collected and organized that evidence into six biological categories and graded each escape route twice: how solid the evidence is in these particular tumors, and whether a drug exists that could counter it. Our aim is to help researchers choose which drug combinations and which biological markers deserve testing first.

## 1. Introduction

### 1.1. Definitions

Neurofibromatosis type 1 (NF1) is the most common hereditary tumor predisposition syndrome and is transmitted in an autosomal dominant manner [1]. The disease arises from mutations in the *NF1* tumor suppressor gene located on chromosome 17q11.2. This gene encodes neurofibromin, a key negative regulator of RAS signaling (predominantly associated with KRAS) [2]. NF1 is characterized by high penetrance and pronounced clinical variability [3,4].

One of the manifestations of NF1 is malignant peripheral nerve sheath tumor (MPNST). These tumors may arise in two ways: as a de novo primary lesion or through a stepwise model of malignant transformation.

Plexiform neurofibromas (PN) are a hallmark of NF1 and occur in 30–50% of patients. They arise during embryonic and early postnatal development and are generally regarded as congenital, although many become clinically apparent only later in childhood as the lesion enlarges. They originate from the proliferation of Schwann cells and other components of the peripheral nerve sheath, forming masses that may be diffuse or localized and may involve several organs and anatomical structures [5]. PN, atypical neurofibromatous neoplasm of uncertain biologic potential (ANNUBP) and MPNST are recognized as successive lesions of a single progression spectrum, but this spectrum is traversed by only a subset of NF1-associated peripheral nerve sheath tumors: PN occur in 30–50% of patients with NF1, whereas the lifetime risk of MPNST is 8–13% (Section 1.2). The great majority of PN therefore never progress, and PN are best described as the lesion from which NF1-associated MPNST most commonly arises rather than as an obligatory precursor stage.

The intermediate lesion of this spectrum is ANNUBP: a pre-malignant neoplasm characterized by loss of neurofibroma architecture, increased cellularity, nuclear atypia and/or mitotic activity that remains below the threshold for malignancy. Its defining molecular event is inactivation of *CDKN2A/B* (encoding p16INK4A and p14ARF), which is already present in the majority of atypical neurofibromas and therefore precedes malignant transformation rather than accompanying it (Section 3.2) [6].

Transformation to MPNST requires further events superimposed on biallelic *NF1* loss and *CDKN2A/B* inactivation. The most characteristic of these is loss of the polycomb repressive complex 2 (PRC2) through inactivation of *SUZ12* or *EED*, which is confined to the malignant stage and is not found in benign or atypical neurofibromas; attenuation of the p53 network is the other recurrent event at this step. Both are considered in detail in Section 3.2 [7].

### 1.2. Epidemiology

According to a 2023 systematic review and meta-analysis (Lee et al.), the pooled prevalence of NF1 is 1 in 3164 individuals (95% confidence interval [CI] 1:2132–1:4712) [8]. The birth incidence is 1 in 2662 newborns (95% CI 1:1968–1:3601). Screening studies reported a substantially higher prevalence (1:2020) than record-based analyses (1:4329), which may indicate underdiagnosis of the disease [8].

In a Finnish population-based registry study [9], the prevalence of NF1 was estimated at 1 in 2052 among individuals aged 0–74 years, with a birth incidence of 1 in 1871. Prevalence was highest in younger cohorts and declined with age, reflecting the increased mortality of patients with NF1 [9].

In a 2025 study by Safonov et al. applying a genotype-first approach to cohorts comprising more than one million individuals, an unexpectedly high frequency of pathogenic *NF1* variants was identified—one case per 1286 individuals [4].

The frequency of plexiform neurofibromas deserves separate mention: they occur in 30–50% of patients with NF1, and in up to 60% when whole-body magnetic resonance imaging is used [1,10].

The lifetime risk of developing MPNST in patients with NF1 is 8–13%, which exceeds the risk in the general population by more than 1000-fold [11,12].

A distinct aetiological category deserves brief mention. A proportion of MPNST arise in a previously irradiated field rather than in the setting of NF1: in a large single-institution clinicopathological series, 13 of 120 tumors (11%) were post-irradiation sarcomas [13], and a systematic review of individual patient data found the prognosis of radiation-induced and radiation-associated MPNST to be worse than that reported for de novo tumors [14]. Molecularly, however, the events on which the resistance mechanisms discussed below rest are largely shared across etiologies: somatic NF1, CDKN2A/B and PRC2 alterations are found in the majority of MPNST irrespective of origin, and PRC2 loss occurs in 90% of radiation-induced, 92% of sporadic and 70% of NF1-associated tumors [15] (Section 3.2). No resistance profile specific to radiation-induced MPNST under MEK inhibition has been described, and no preclinical or clinical data indicate that these tumors respond to MEK inhibition differently from NF1-associated ones. Because radiation-induced MPNST largely fall outside the NF1-associated PN → ANNUBP → MPNST spectrum that is the subject of this review, they are not treated as a separate category in Section 3.5.

### 1.3. MEK Inhibitors as the Current Therapeutic Standard

MEK inhibitors (selumetinib, mirdametinib) constitute the only approved class of targeted agents for NF1-associated plexiform neurofibromas [16].

In adults with inoperable PN, a confirmed partial response was achieved in 63.6% of cases [17]. In the KOMET trial, the objective response rate with selumetinib was 19.7%, significantly higher than the 5.4% recorded with placebo [18]. In MPNST, by contrast, single-agent MEK inhibition has proved clinically ineffective [19]; for this reason, comparable response data are not available.

These figures are not directly comparable, and the distance between them is in large part methodological rather than biological. The two adult studies differ in design and, critically, in who adjudicated the response: the single-arm phase 2 trial counted confirmed partial responses accruing over prolonged treatment in a small cohort [17], whereas KOMET counted objective responses established by independent central review according to the Response Evaluation in Neurofibromatosis and Schwannomatosis (REiNS) criteria by cycle 16 [18]. The size of that adjudication effect is documented within a single dataset: in SPRINT, the registrational pediatric trial of selumetinib, 34 of 50 children (68%) had a confirmed partial response on investigator assessment but 44% (95% confidence interval 30–59) on blinded independent central review of the same scans [20]. The only comparison of the two age groups made within one protocol and by one method comes from ReNeu, in which mirdametinib was assessed by blinded independent central review in both: 24 of 58 adults (41%) and 29 of 56 children (52%) achieved a confirmed objective response, with a median best volumetric reduction of 41% and 42% respectively [21]. Children therefore respond somewhat more often than adults, but by a margin of roughly ten percentage points rather than the threefold gap suggested by the raw figures; the residual difference may reflect the higher growth rate of plexiform neurofibromas in younger patients, although this has not been tested formally. For the present review, the consequence is practical: the depth and duration of response, and therefore the point at which a tumor is judged refractory, are age-dependent, so resistance data obtained in pediatric cohorts should not be assumed to transfer to adults or the reverse.

### 1.4. Rationale and Aim of the Review

The existing literature consists of several fragmented primary studies, each describing an individual resistance mechanism in a particular tumor type, together with general reviews of NF1 therapy in which resistance is mentioned only as one of several unresolved problems. No comprehensive literature review describing the mechanisms of resistance to MEK inhibitors in MPNST and its precursor lesions is currently available. The present work therefore contributes to the development of antitumour pharmacotherapy aimed at improving treatment efficacy. Armstrong et al. (2026) state explicitly that potential MEK inhibitor resistance remains underexplored [5], and Wang et al. (2022) note that few studies have investigated the mechanisms of MEK inhibitor resistance in patients with PN [22].

The aim of this review was to systematize the established molecular mechanisms of resistance to MEK inhibitors in NF1-associated plexiform neurofibromas and MPNST, together with biologically plausible candidate mechanisms extrapolated from other RAS-driven tumors that require formal validation.

## 2. Materials and Methods

This article is a narrative literature review. The selection algorithm comprised search, removal of duplicates, screening by title and abstract, full-text assessment, and formation of the final list, and the search strategy is reported below in full so that it can be reproduced independently. Ninety-six sources met the criteria and are cited; record counts at the intermediate selection steps were not maintained, and no PRISMA flow diagram is presented.

Sources. PubMed, PubMed Central, NCBI Bookshelf and Scopus were searched. These were supplemented by clinical practice guidelines, official regulatory documents (product labels and the FDA Orange Book) and by hand-searching the reference lists of the retrieved articles.

Dates and coverage. The initial search covered publications available up to 31 May 2026 and was repeated with identical search strings in August 2026 in order to capture work appearing during peer review; this update identified six additional primary studies, which have been incorporated into the relevant subsections of the Results and Conclusions. No lower date limit was applied, so that foundational work on the biochemistry of the RAS–MAPK cascade could be included. No language restriction was applied a priori, and all but one of the included publications are in English. A small number of further sources cited in Section 3.5.1 and Section 4—concerning the terminology of on-target resistance, the mechanism of RAF-mediated MEK phosphorylation and existing frameworks of clinical actionability—were identified by targeted searching during revision rather than by the strings below.

Search strings. The following combinations were used, with automatic term mapping enabled: (i) (“neurofibromatosis type 1” OR NF1) AND (“MEK inhibitor” OR selumetinib OR mirdametinib OR trametinib); (ii) (“neurofibromatosis type 1” OR NF1 OR “plexiform neurofibroma” OR “malignant peripheral nerve sheath tumor” OR MPNST) AND (resistance OR refractory OR relapse OR “treatment failure”); (iii) (“MEK inhibitor” OR “MAPK pathway”) AND resistance AND (“negative feedback” OR “RAF dimerization” OR SHP2 OR SOS1 OR “receptor tyrosine kinase” OR PI3K OR autophagy OR “tumor microenvironment” OR “single-cell”); (iv) (“plexiform neurofibroma” OR MPNST) AND (SUZ12 OR EED OR PRC2 OR CDKN2A OR TP53); and (v) for every candidate mechanism retrieved by (iii), a targeted string combining the mechanism term with (NF1 OR MPNST OR “plexiform neurofibroma”), in order to establish whether direct evidence existed in these entities or only in other tumor types.

Inclusion criteria. Original experimental studies (in vitro, in vivo and ex vivo), clinical trials and their secondary analyses, meta-analyses, and current clinical practice guidelines. Reviews were used for orientation, for cross-referencing and for background and definitional statements only: no factual claim in this article rests on a review as its sole source, and wherever a finding is reported, the primary study was retrieved and cited.

Exclusion criteria. Records not concerned with NF1-associated nerve sheath tumors or with MEK/MAPK-directed therapy; duplicate records; conference abstracts and preprints that had not undergone peer review; publications reporting neither primary data nor a traceable primary source; and publications in which the methods reported were insufficient to verify the result being cited.

Because the mechanisms discussed rest on very different bodies of evidence, each was annotated with the entity and model in which it had been demonstrated—PN, MPNST, another NF1-deficient tumor, or an NF1-wild-type RAS-driven tumor of a different lineage—and was then graded along two independent axes, the strength of evidence in PN or MPNST and the therapeutic tractability. The grading criteria are defined at the beginning of Section 4 and applied in Table 1.

**Figure 1:**
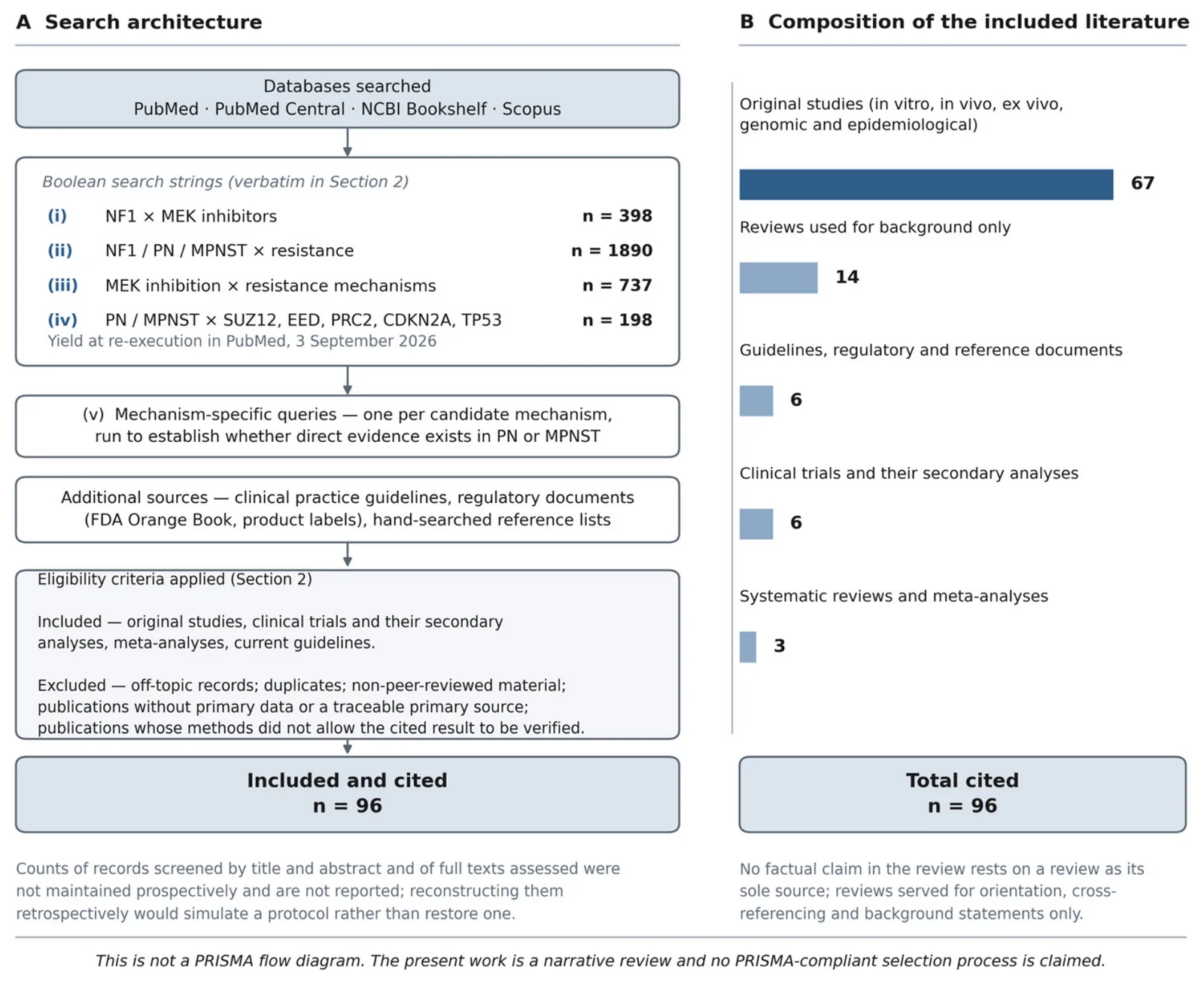
Search architecture and composition of the included literature. Left: the four Boolean search strings, the mechanism-specific fifth stage, and the supplementary sources (guidelines, regulatory documents, hand-searched reference lists), with the yield of each Boolean string at re-execution in PubMed on 3 September 2026. Right: the composition of the 96 cited sources by publication type (67 original studies; 6 clinical trials or secondary analyses; 3 systematic reviews or meta-analyses; 6 guidelines, regulatory or reference documents; 14 reviews used for background only).

Search yield and composition of the included literature. The four Boolean strings above were re-executed in PubMed on 3 September 2026, returning 398, 1890, 737 and 198 records respectively; these figures are reproducible by any reader entering the strings exactly as printed. The numbers of records screened by title and abstract and of records assessed in full text were not counted prospectively and are therefore not reported: reconstructing them retrospectively would simulate a protocol rather than restore one. The composition of the literature that was included is, by contrast, exact and verifiable line by line against the reference list. Of the 96 cited sources, 67 are original studies (in vitro, in vivo, ex vivo, genomic and epidemiological), 6 are clinical trials or their secondary analyses [17,18,20,21,46,49], 3 are systematic reviews or meta-analyses, 6 are clinical practice guidelines, regulatory documents or reference sources, and 14 are reviews used for orientation and background only. This architecture is summarized in Figure 1. The figure is deliberately not presented as a PRISMA flow diagram: the present work is a narrative review, screening counters were not maintained, and no PRISMA-compliant selection process is claimed.

The strength of evidence was graded on an explicit axis constructed for this review (E1–E3, defined at the beginning of Section 4), which grades the question actually at issue: whether a mechanism has been demonstrated in PN or MPNST, and whether that demonstration included a functional perturbation experiment.

The re-execution reported here was performed to document the reproducible yield of each string and does not constitute a further round of screening; the search update that added new sources to the review was the August 2026 repetition described in Section 2. This is not a PRISMA flow diagram: the present work is a narrative review, counts of records screened by title and abstract and of full texts assessed were not maintained prospectively, and they are not reconstructed here.

No generative artificial intelligence was used to generate scientific content, data, or interpretations; its use was limited to language editing.

## 3. Results

### 3.1. Biochemical Basis of NF1

**Figure 2:**
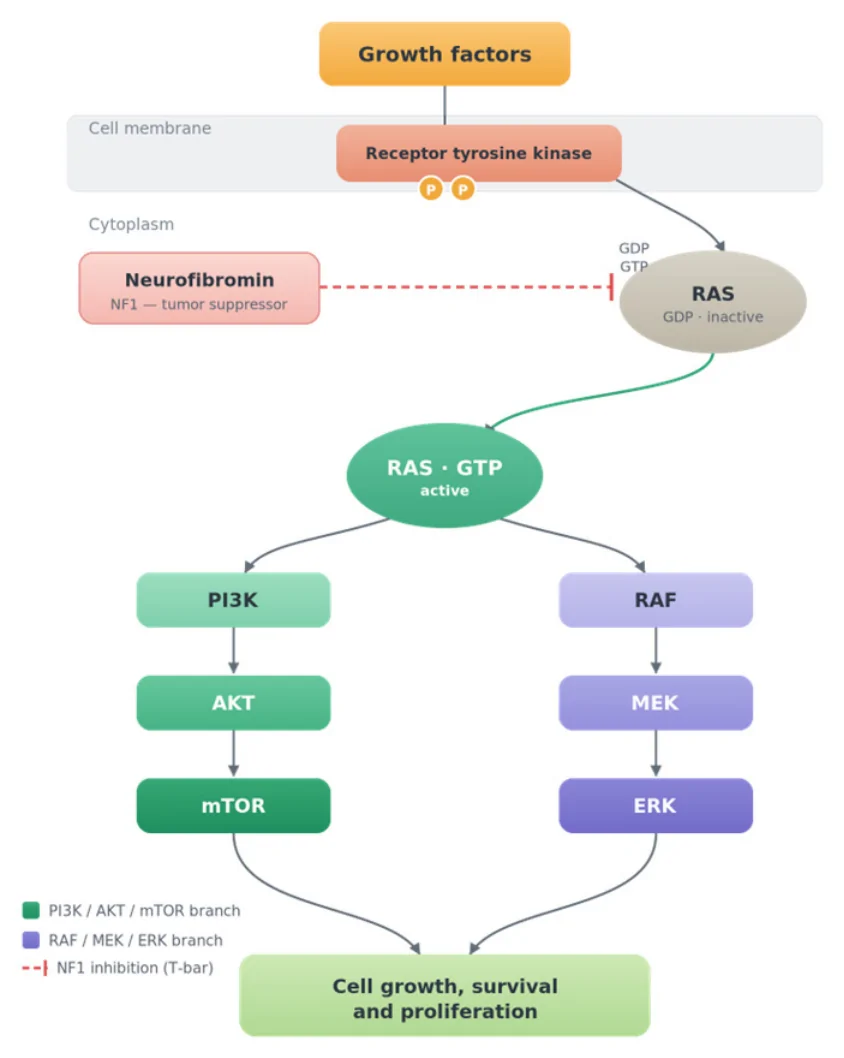
RAS signaling in the presence of defective neurofibromin.

The pathogenesis of NF1 is linked to the RAS–MAPK signaling pathway, which transmits signals from the cell surface to the nucleus and regulates fundamental processes including proliferation, differentiation, survival, apoptosis and migration. Attenuation of this biochemical cascade, and hence of proliferative activity, is achieved by hydrolysis of RAS-GTP (the active form) to RAS-GDP (the inactive form). This reaction is catalyzed by neurofibromin, the key negative regulator of RAS signaling encoded by the *NF1* tumor suppressor gene on chromosome 17q11.2 [1,2]. In performing this function, neurofibromin acts as a RAS GTPase-activating protein (RAS-GAP): it accelerates the intrinsically slow hydrolysis of RAS-GTP by several orders of magnitude. Accordingly, mutations in this gene result in the synthesis of a defective neurofibromin, loss of its RAS-GAP activity, accumulation of RAS in the GTP-bound state, and persistent RAS activation [1,2]. The inability to switch off the pathway leads to hyperactivation of two key cascades (Figure 2):

• The RAS → RAF → MEK → ERK axis (the MAPK cascade): ERK phosphorylates nuclear transcription factors, thereby driving cell proliferation and differentiation [2];
• The RAS → PI3K → AKT → mTOR axis: loss of neurofibromin results in constitutive mTOR activation through AKT-mediated phosphorylation and inactivation of tuberin (TSC2), which stimulates cell growth [83].

### 3.2. Genetic Landscape

Inactivation of *NF1* alone is insufficient for malignant transformation; progression to sarcoma requires the accumulation of additional genetic events. This mode of tumor development corresponds to Knudson’s two-hit model. Tumor formation requires inactivation of the second allele in somatic cells, resulting in complete loss of functional neurofibromin [2]. Neurofibromin not only exerts the RAS-GAP activity described in Section 3.1 but also regulates adenylate cyclase and intracellular cyclic adenosine monophosphate (cAMP) levels, and influences cell motility through the RHO/ROCK pathway [2].

**Figure 3:**
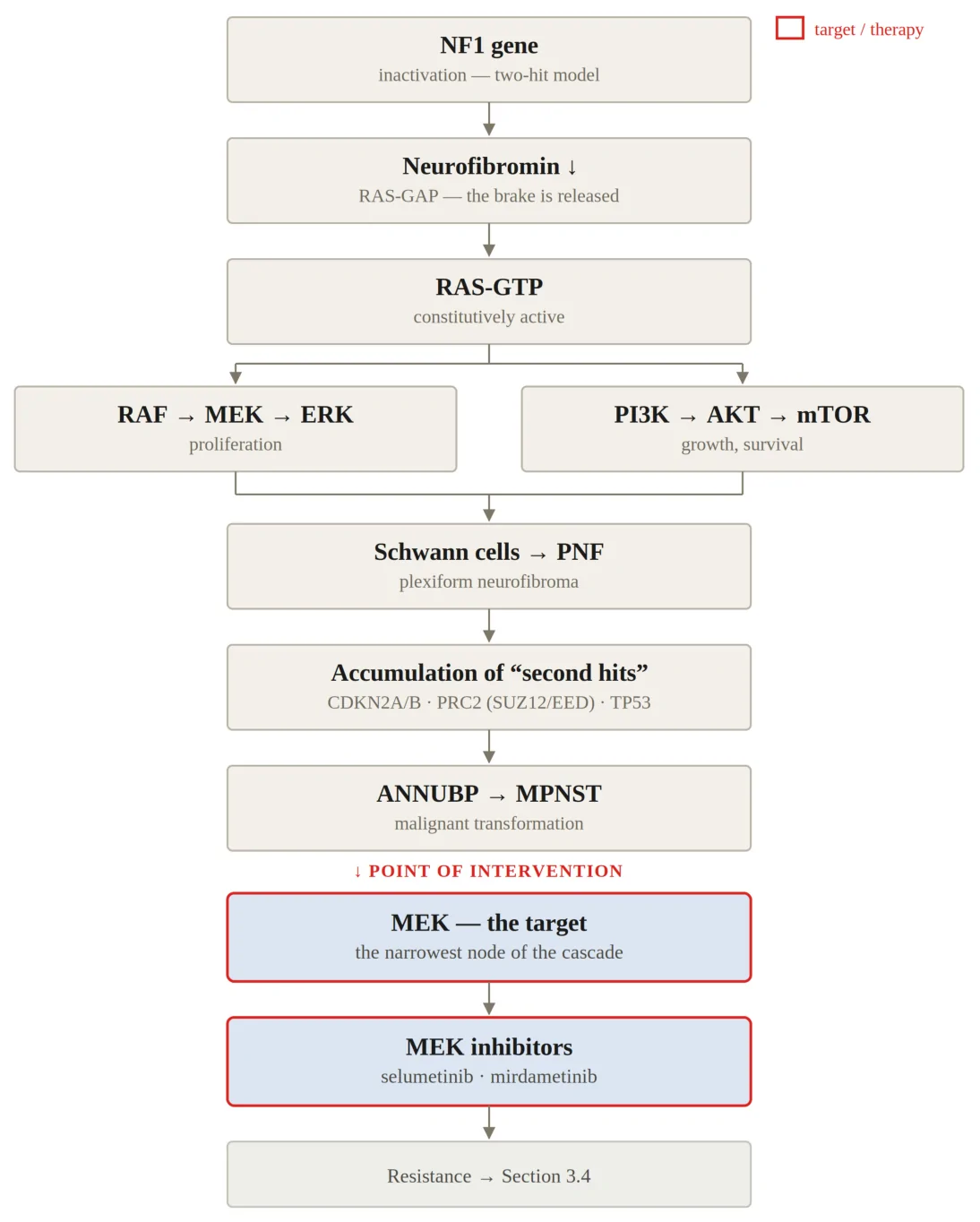
Integrated scheme of the successive stages of MPNST formation.

In addition to *NF1*, MPNST harbors a number of recurrent gene alterations, each with a defined role in pathogenesis, diagnosis and prognosis:

1. Homozygous deletion, translocation or point mutation of *CDKN2A/B* is the earliest genetic event in the transition from benign neurofibroma to MPNST. Deletion of this locus is already detectable in atypical neurofibromas at a frequency of approximately 69%, indicating that *CDKN2A/B* inactivation may be the principal event driving pre-malignant transformation [84]. From a therapeutic perspective, *CDKN2A*-deleted MPNSTs show increased dependence on the pentose phosphate pathway and NADPH metabolism, which opens new avenues for the development of targeted strategies [85].
2. Inactivation of PRC2 components (SUZ12 or EED) is the defining molecular event of malignant transformation in MPNST and is absent from benign and atypical neurofibromas. The frequency of PRC2 loss varies by subtype: 92% in sporadic, 70% in NF1-associated, and 90% in radiation-induced MPNST [15]. A detailed description of the complex, the functional consequences of PRC2 loss, and the associated resistance mechanism is provided in Section 3.5.3.
3. Complete, biallelic inactivation of *TP53*—a point mutation accompanied by loss of heterozygosity—is uncommon, occurring in 8.2% of MPNST, with MDM2 amplification accounting for a further 5.5% in a mutually exclusive manner [86]. Taken in isolation, this frequency would not justify placing *TP53* alongside *CDKN2A/B* and PRC2. Restricting the analysis to complete loss of function, however, substantially underestimates involvement of the pathway. When partial loss of function is included—copy-number aberration and/or loss of heterozygosity at the *TP53* locus in the absence of a point mutation—the affected tumors converge on a common transcriptional “*TP53*-mutated phenotype” that is detectable in as many as 60% of MPNST and defines a subgroup with markedly inferior five-year disease-specific survival (hazard ratio 4.1, 95% confidence interval 1.7–9.8) [86]. It is this graded, dose-dependent attenuation of the *p53* network, rather than complete biallelic loss, that gives *TP53* a standing comparable to that of *CDKN2A/B* and PRC2, and that underpins its treatment as a resistance mechanism in Section 3.5.3 and its classification in Table 1. The successive stages of this progression, from biallelic NF1 inactivation through atypical neurofibroma to MPNST, are summarized in Figure 3.

### 3.3. Pharmacodynamics of MEK Inhibitors

Despite the heterogeneity of secondary alterations (*CDKN2A/B*, PRC2, *TP53*), all of them are superimposed on a common initiating event—hyperactivation of the RAS–RAF–MEK–ERK axis. It is for this reason that MEK, as the narrowest and most pharmacologically tractable node of the cascade, was selected as the therapeutic target.

Agents that suppress MEK activity in NF1 are selective allosteric modulators of MEK1/2. They block the phosphorylation of ERK1/2, the principal effector of the hyperactive RAS/RAF/MEK/ERK cascade caused by loss of neurofibromin function [87]. Two agents are currently approved by the U.S. Food and Drug Administration (FDA) for NF1-associated plexiform neurofibromas: **selumetinib** and **mirdametinib** [16].

**Figure 4:**
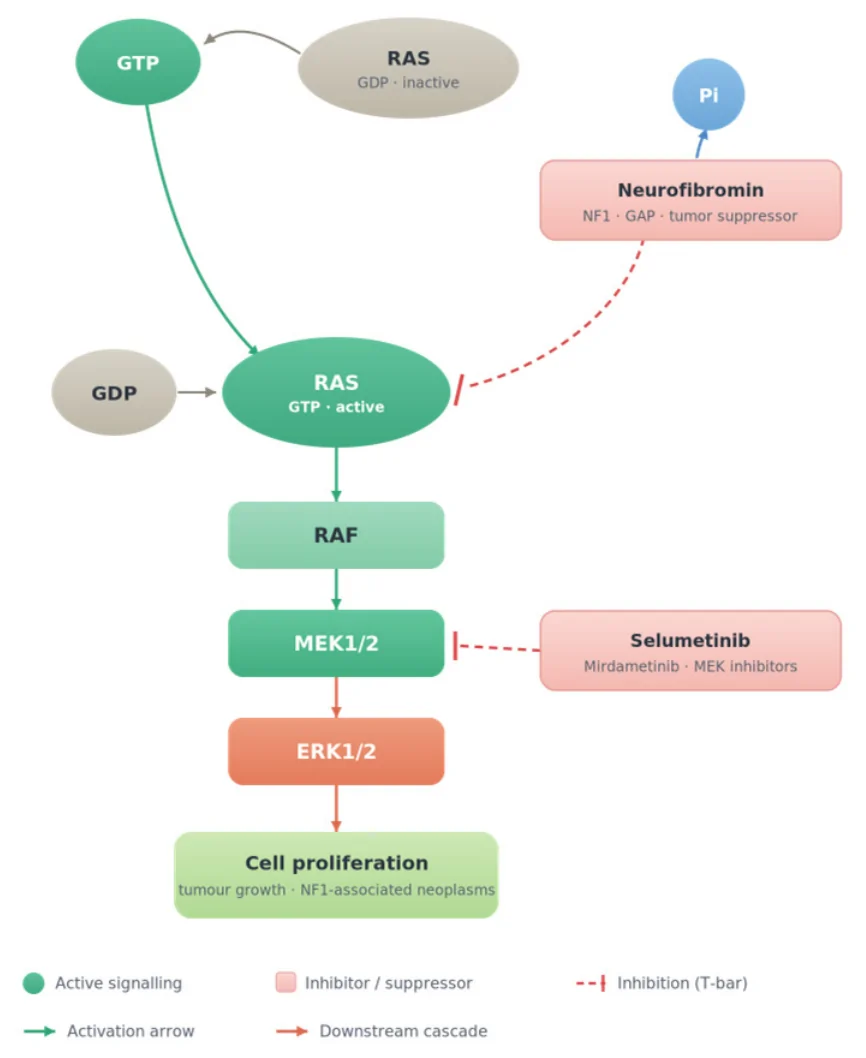
Mechanism of action of MEK inhibitors.

Following MEK inhibition, ERK phosphorylation declines, reducing the transcription of proliferation-associated genes and suppressing the proliferative activity of tumor cells. Both agents are used as targeted therapy [88]. The sequence of events from allosteric MEK1/2 blockade to suppression of proliferative activity is summarized in Figure 4.

### 3.4. Pharmacological Differences Between MEK Inhibitors

Mirdametinib has a substantially longer half-life (approximately 28 h versus approximately 9 h), a fundamentally different metabolic route and superior blood–brain barrier penetration. It also displays higher biochemical potency: the half-maximal inhibitory concentration (IC_50_) for MEK1 is approximately 1.9 nM, compared with approximately 14 nM for selumetinib, making mirdametinib an approximately sevenfold more potent MEK1 inhibitor in vitro [87].

Selumetinib, because of its short half-life, requires uninterrupted daily administration to maintain therapeutic concentrations [87].

Selumetinib is metabolized predominantly via CYP3A4 (oxidation) and UGT-mediated glucuronidation; co-administration of strong CYP3A4/CYP2C19 inhibitors or inducers alters its exposure to a clinically meaningful extent and requires corresponding dose adjustment. Mirdametinib shows a fundamentally different metabolic profile: it is cleared mainly through glucuronidation by UGT1A6 and UGT2B7 and by carboxylesterases (CES), without substantial CYP involvement, which confers a lower potential for CYP-mediated drug–drug interactions. Mirdametinib is, however, a substrate of the P-glycoprotein (P-gp) and BCRP transporters, and P-gp inhibition could theoretically increase its absorption, although in the absence of inhibitors the drug is rapidly and completely absorbed owing to its high permeability and solubility [89].

Mirdametinib therefore carries a considerably lower potential for CYP-mediated drug interactions, which simplifies its concomitant use with other medicinal products.

Notwithstanding these pharmacological differences, both agents share a common limitation: the response to single-agent MEK inhibition is restricted in both depth and duration. Tumors reactivate MAPK signaling and engage bypass routes, and it is precisely these resistance mechanisms that define the limits of targeted therapy. Their systematization is the subject of the following section.

### 3.5. Mechanisms of Resistance to MEK Inhibitors

For the purposes of systematization we adapted the convergent framework of resistance to targeted therapy proposed by Konieczkowski et al. [90], which reduces heterogeneous mechanisms to a limited number of convergence points: reactivation of the blocked pathway, its bypass, and the emergence of a state indifferent to the target. This “level of execution” axis was superimposed on the classical division of resistance into primary, adaptive, and acquired. In contrast to the original frameworks, which were developed largely in BRAF-mutant melanoma models, we applied them to NF1-associated plexiform neurofibroma and MPNST and expanded the “indifference” category into discrete levels—epigenetic rewiring, cell survival programs, the tumor microenvironment and immunity, and intratumoural heterogeneity—that reflect the specific biology of these entities. A synoptic map of all mechanisms discussed below is provided as Figure S1.

Descriptively, all resistance mechanisms can be characterized along two axes: the locus or level at which they operate, and the type of resistance (primary, adaptive or acquired). These axes describe where and how a mechanism acts; the separate questions of how firmly it is evidenced in PN or MPNST and how tractable it is therapeutically are addressed in Section 4.

Primary resistance arises de novo. Adaptive resistance (rapid, gene-independent) develops within hours to days and has been described for MAPK cascade inhibitors by Lito et al. [91]; it is non-genetic and reversible. Acquired resistance (slow, gene-dependent) develops over weeks and becomes genetically fixed.

Each mechanism below closes with a standardized annotation stating the type of resistance, the evidence source—the entity and the model in which the mechanism was demonstrated—and the two grades applied in Table 1: the strength of evidence that the mechanism confers resistance to MEK inhibition in PN or MPNST (E1–E3) and the therapeutic tractability of the mechanism (T1–T3). The grading criteria are defined at the beginning of Section 4. The same annotations are collated for all mechanisms in Table S1, which follows the order of Section 3.5.1, Section 3.5.2, Section 3.5.3, Section 3.5.4, Section 3.5.5 and Section 3.5.6 and additionally states the experimental method in each case.

#### 3.5.1. Reactivation of MAPK Signaling Within the Cascade (Pathway-Dependent Resistance)

In this category, flux through the cascade is restored despite continued drug exposure by events located within the RAS–RAF–MEK–ERK axis itself—upstream of MEK or at its regulators. We deliberately do not describe these mechanisms as “on-target”. That term is reserved here for alterations of the drug target proper, namely activating mutations of *MAP2K1*/*MAP2K2*, which reduce inhibitor binding or render MEK constitutively active; such alterations underlie acquired resistance to MAPK-directed therapy in BRAF-mutant melanoma [92], but have not so far been reported in PN or MPNST progressing on selumetinib or mirdametinib. Their absence from this review reflects the absence of data rather than a demonstrated absence of the phenomenon, and sequencing of *MAP2K1*/*MAP2K2* at progression would be a straightforward addition to current clinical practice.

One of the key resistance mechanisms is disruption of negative feedback within the RAS/RAF/MEK/ERK cascade. In the untreated state, activated ERK restrains upstream components of the cascade by phosphorylating SOS1, suppressing RAF expression, and inducing DUSP and Sprouty (SPRY) proteins. This creates a brake that limits excessive pathway activation [59].

When MEK is inhibited, ERK activity falls, ERK-dependent restraint of RAF/RAS is lost, and several parallel compensatory events are triggered that restore signal flux through the cascade:

• *RAF dimerization*

Upon RAS activation, recruitment of RAF to the membrane is enhanced: GTP-bound RAS binds RAF and promotes removal of inhibitory phosphosites (for example, Ser259 in CRAF) through the SHOC2/PP1 complex [23].

MEK inhibition in MPNST cells induces increased formation of RAF dimers. Because RAF lies upstream of MEK, this does not bypass MEK: dimerized RAF sustains phosphorylation of the MEK activation loop, and allosteric MEK inhibitors of the selumetinib and mirdametinib class do not, or only incompletely, prevent this RAF-mediated phosphorylation, so that flux through MEK is partially restored despite inhibitor occupancy [93]. Consistent with this, combining a MEK inhibitor with RAF dimer inhibitors produced pronounced synergistic suppression of MAPK signaling both in vitro and in vivo [24].

The SHOC2/PP1 complex is a holophosphatase that dephosphorylates the inhibitory site on RAF kinases, a step required for RAF dimerization [25]. Functional genomic studies of NF1-deficient tumors identified SHOC2, alongside BRAF, as a mediator of the response to MEK inhibition both in vitro and in vivo [26]. Genetic inhibition of SHOC2 prevents MEK inhibitor-induced RAF dimerization and thereby sustains suppression of the ERK pathway.

In essence, ERK-mediated phosphorylation of BRAF and CRAF (RAF isoforms capable of forming homo- and heterodimers) acts through negative feedback to stabilize the inactive conformation and to prevent dimerization [23].

Type: adaptive—allosteric MEK inhibition relieves feedback, rapidly increasing the number of active RAF dimers that sustain phosphorylation of the MEK activation loop and thereby restore flux through MEK despite inhibitor occupancy, in a gene-independent manner. Model: NF1-deficient MPNST (mouse); in vivo [24]. Evidence source: NF1-deficient MPNST (murine models and cell lines); in vivo and in vitro. Grade: E1/T1.

• *Amplification or mutation of the genes encoding NRAS/KRAS*

Prolonged exposure to MEK inhibitors may become genetically fixed through amplification or mutation of driver oncogenes.

In experimental models of colorectal cancer (a tumor of different origin) harboring BRAF V600E or KRAS G13D mutations, prolonged exposure to selumetinib led to an increase in the copy number of the corresponding oncogene on a single chromosome. This enhanced signal transmission through the RAF → MEK → ERK cascade and restored ERK1/2 activity to the level required for cell growth and division [25].

Type: **acquired**—it emerges only after prolonged MEK inhibitor exposure through selection of clones carrying genetic events. Model: *BRAF-mutant melanoma and RAS-mutant colorectal cancer—NF1-wild-type tumors of non-nerve-sheath lineage*; in vitro [25,74]. Evidence source: NF1-wild-type tumor cell lines of non-nerve-sheath lineage (melanoma, colorectal cancer); no data in PN or MPNST. Grade: E3/T3.

• *Dissolution of P-bodies*

MEK inhibitors also increase the synthesis of KRAS and NRAS proteins by activating a distinct mechanism linked to the dissolution of P-bodies—cytoplasmic structures that sequester RAS messenger RNA. MEK suppression leads to disassembly of P-bodies, releasing KRAS/NRAS transcripts and triggering rapid feedback reactivation of MAPK signaling. This hypothesis is based on cellular (in vitro) models derived from several tumor types: lung adenocarcinoma, melanoma and breast cancer [75].

Type: **adaptive**—MEK inhibition dissolves P-bodies, releasing messenger RNA and increasing RAS protein synthesis; the authors explicitly demonstrate reversibility upon drug withdrawal [75]. Evidence source: NF1-wild-type tumor cell lines of non-nerve-sheath lineage; in vitro. Grade: E3/T3.

These two mechanisms (amplification or mutation of *NRAS/KRAS*, and dissolution of P-bodies)—despite their different nature, the former acquired and the latter adaptive—have so far been established only in NF1-wild-type tumor cell lines of non-nerve-sheath lineage. Two independent gaps are therefore involved, and they are distinguished consistently throughout this review. The first is genetic background: in these models neurofibromin is intact, so RAS output remains buffered by residual GAP activity, whereas in PN and MPNST, that buffer is absent and the same perturbation may have a quantitatively different consequence. The second is lineage and context: Schwann cell biology and the stroma-rich microenvironment of PN and MPNST are not represented. For the two mechanisms discussed here, both gaps apply simultaneously, which is why they are presented as promising hypotheses rather than as proven resistance routes in peripheral nerve sheath tumors.

• *Loss of DUSP/SPRY-mediated feedback*

DUSPs (dual-specificity phosphatases) and Sprouty (SPRY) proteins are ERK-associated proteins that inactivate the entire signaling pathway through negative feedback within the MAPK cascade.

DUSPs dephosphorylate the activation loop of ERK1/2, providing fine spatiotemporal control of MAPK signaling. SPRY acts further upstream, at the RTK → RAS → RAF level, suppressing RAS activation.

Because both are ERK-inducible gene products, these proteins are markedly downregulated upon MEK inhibition, which releases negative feedback and promotes reactivation of the cascade [60].

Type: **adaptive**—DUSP and SPRY are ERK-inducible: when ERK activity falls, they disappear within hours, the brake is released without any mutation, and the effect is reversible. Model: NF1 peripheral nervous system atlas plus MPNST cell lines; in vivo/in vitro [59,61]. Evidence source: NF1-associated peripheral nerve tissue atlas and MPNST cell lines; expression-level and pharmacodynamic data, without a rescue experiment. Grade: E2/T3.

• Convergence of bypass inputs on SHP2 (PTPN11) → SOS1

**Figure 5:**
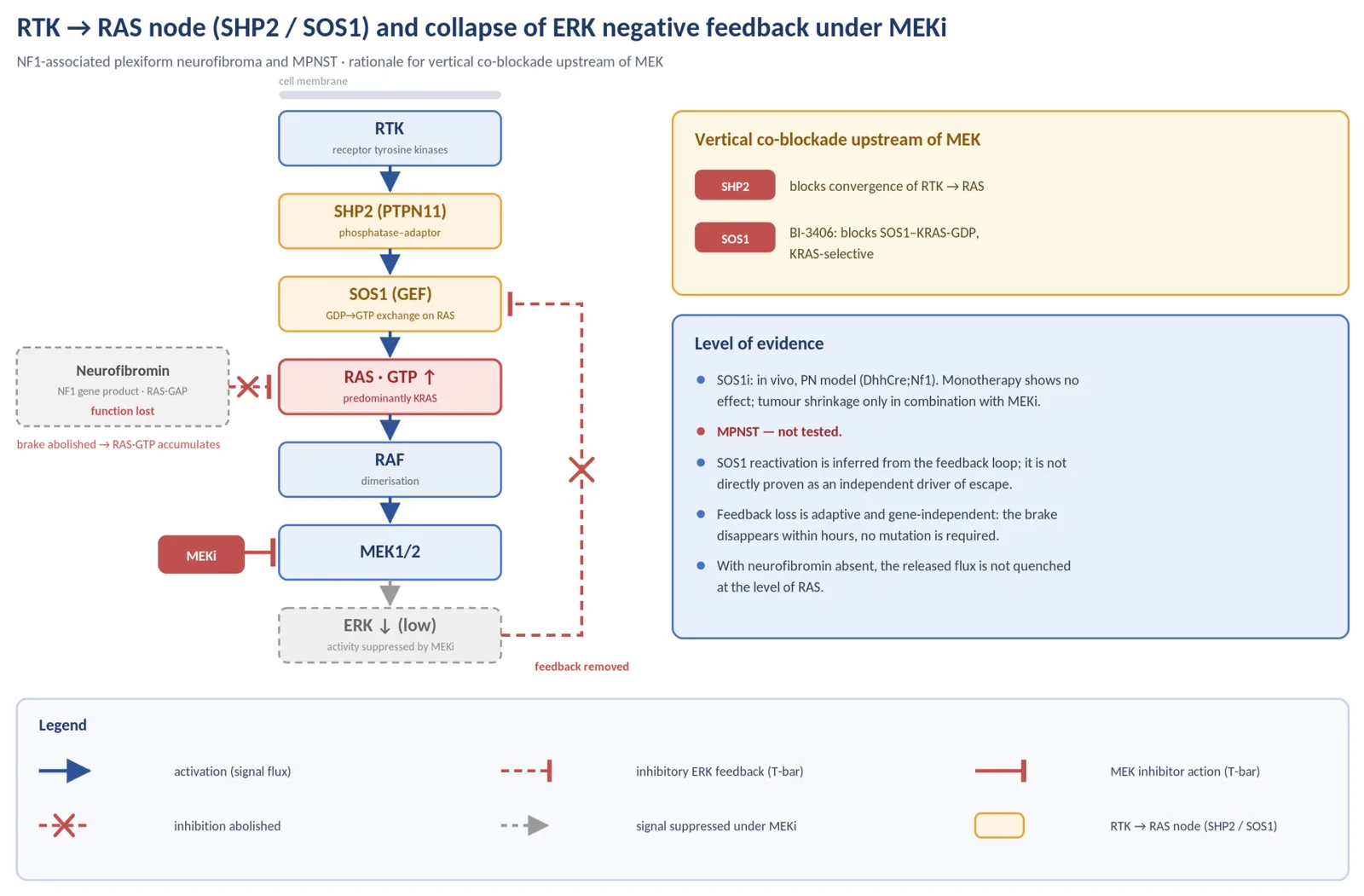
Disruption of ERK-dependent negative feedback under MEK inhibition.

Point of convergence: the events described above—RAF dimerization, RAS recruitment and release of SPRY-mediated restraint—converge on the SHP2 (PTPN11) → SOS1 node, which relays signals from receptor tyrosine kinases to RAS. Vertical co-inhibition of this node, in particular the combination of a MEK inhibitor with SHP2 inhibitors [27], therefore blocks several reactivation branches simultaneously (Figure 5).

Type: **adaptive**—global RTK reactivation after MEK inhibition rapidly converges on SHP2 as the common node upstream of RAS. Model: NF1-deficient MPNST (mouse and cell lines); in vivo [27]. Evidence source: NF1-deficient MPNST (cell lines and murine models), with an SHP2 inhibitor–MEK inhibitor combination tested in vivo. Grade: E1/T1.

Jackson et al. (2023) additionally considered SOS1 as a rational vertical co-target upstream of RAS. Mechanistically, it is linked to reactivation through negative feedback, but it has not been proven to act as an independent driver of escape [28]. Complementary to these hypothesis-driven vertical strategies, unbiased pharmacogenomic synthetic-lethal screening across NF1-deficient models has approached the same problem from the opposite direction: digoxin and rigosertib were selectively active against NF1-null cells, synergized with selumetinib and—directly relevant to the mechanism discussed here—reduced the feedback reactivation of RAS that follows MEK blockade [29].

#### 3.5.2. Parallel (Bypass) Reactivation of MAPK Through Adjacent Inputs (Pathway-Independent)

In this category, signaling is restored through receptor tyrosine kinases and parallel effectors.

• *Receptor tyrosine kinases (RTKs)*

RTK reactivation is the most reproducible class of resistance to MEK inhibitors and has been described predominantly in MPNST. Its driving force is the release of ERK-dependent negative feedback: MEK blockade abolishes physiological suppression of upstream components (RTKs, SOS1, RAF) and of the DUSP/SPRED phosphatases, disinhibiting the receptor pool and restoring RAS-GTP flux around the point at which the drug acts. In the setting of biallelic *NF1* inactivation, this signal is not quenched at the level of RAS, because the GAP function of neurofibromin is lost.

In MPNST, the role of the HGF/MET axis has been demonstrated: HGF overexpression confers resistance on initially sensitive cells, and silencing of HGF or MET reverses it [30]. A second independently confirmed RTK axis is transcriptional upregulation of PDGFRβ in response to MEK blockade [24]. Both observations reflect a common principle: MEK blockade releases ERK-dependent feedback and disinhibits receptor signaling, which is relayed downstream through the cytoplasmic tyrosine phosphatase SHP2 (PTPN11)—the proximal RAS–MAPK node between RTKs and RAS. An indirect indication of the importance of this node in MPNST is that reducing SHP2 activity does not abolish escape but shifts it to a downstream survival level, with activation of protective autophagy [31], illustrating the sequential character of resistance.

In PN, convincing evidence for RTK reactivation as an independent mechanism of resistance to MEK inhibitors is currently lacking. The single study in which an IGF-1R inhibitor was tested in PN was methodological in nature (development of a three-dimensional plexiform neurofibroma model) and demonstrated not receptor reactivation but a non-specific reduction in cell sensitivity to all agents tested, including selumetinib, under three-dimensional culture conditions [32].

The entire causal evidence base derives from selected preclinical models (cell lines, xenografts); direct evidence of RTK reactivation in material from patients progressing on MEK inhibitors is absent from the literature. Priority directions are confirmation of RTK/SHP2 reactivation in paired biopsies obtained before treatment and at progression, and clarification of the relevance of RTK reactivation specifically in PN.

**Type: adaptive and acquired.** The adaptive component is the immediate, gene-independent, and reversible disinhibition of the RTK pool through release of ERK-dependent negative feedback (hours to days). The acquired component is stable HGF/MET dependence, fixed in selected resistant MPNST lines: HGF overexpression confers resistance on initially sensitive cells, and suppression of HGF or MET reverses it. This is precisely how the primary source (Wang et al., 2021) defines this class. Model: NF1-deficient MPNST lines selected in vitro. Evidence source: MPNST (resistant cell lines with HGF/MET silencing and rescue; murine models). Grade: E1/T1.

• *Integrin signaling*

**Figure 6:**
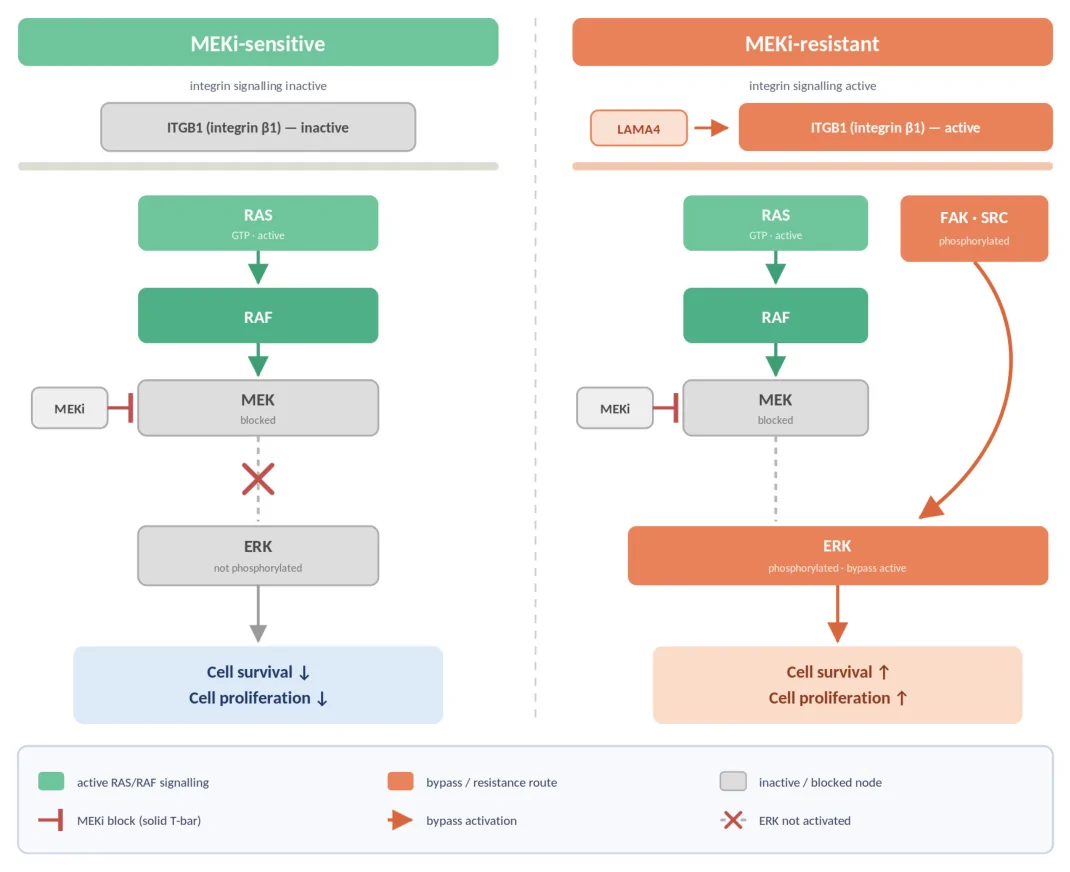
The LAMA4/ITGB1/FAK/SRC cascade in MEK inhibitor-resistant MPNST cells.

In MEK inhibitor-resistant MPNST cells, one of the key mechanisms for bypassing MAPK pathway blockade is activation of integrin-mediated adhesion signaling: LAMA4 → ITGB1 → FAK → SRC. This axis allows tumor cells to reactivate the proliferative signaling cascade even under MEK inhibition [44]. In effect, the tumor grows not only through MAPK signaling but also through signals from its microenvironment. The mechanism comprises the following steps: increased expression of LAMA4 (laminin α4, a component of the extracellular matrix) → activation of integrin β1 (ITGB1) → autophosphorylation of FAK (focal adhesion kinase) → formation of the FAK/SRC complex → activation of the MAPK/ERK and PI3K/AKT pathways [44] (Figure 6).

Type: **acquired**—the adhesion-dependent survival signal becomes fixed specifically in *resistant* MPNST cells. Model: resistant MPNST lines; in vitro [44]. Evidence source: resistant MPNST cell lines; in vitro only. Grade: E2/T2.

• *Loss of NF2, PAK activation and Schwann cell dedifferentiation*

Merlin, the product of *NF2*, is a broadly acting tumor suppressor that normally inhibits PAK1/2 (p21-activated kinases) and also governs contact inhibition and the Hippo/YAP-TAZ pathway. In a functional genomic screen of NF1-mutant cells, suppression of *NF2* emerged as one of the strongest drivers of resistance to selumetinib [26]. Loss of NF2 releases PAK from inhibition, driving cells into a less differentiated state that sustains proliferation independently of MEK inhibition; combined loss of *NF1* and *NF2* defines a distinct dedifferentiated subgroup of tumors. At the molecular level, activated PAK can sustain RAF/ERK signaling (through phosphorylation of RAF-S338 and MEK-S298), but the key resistance mechanism here is lineage plasticity rather than direct reactivation of the cascade; the mechanism was therefore assigned according to its dominant feature. Combined MEK and PAK inhibition restored sensitivity in xenograft models [26].

Type: **primary/adaptive**—loss of the Merlin suppressor is the initial structural event (the primary component) in Schwann cell dedifferentiation, whereas PAK disinhibition operates as a network-level adaptation. Model: MPNST (dedifferentiation); in vivo/in vitro [26]. Evidence source: MPNST and PN (cell lines and in vivo); TEAD-inhibitor resensitization demonstrated in two MPNST lines in vitro. Grade: E2/T2.

The roles of contact inhibition and the Hippo/YAP-TAZ pathway merit description for a fuller understanding of this resistance mechanism.

Contact inhibition of proliferation is a physiological mechanism whereby normal cells cease dividing once a confluent monolayer is reached, receiving the signal through intercellular junctions. This is mediated by Merlin, which is dephosphorylated (the active form), concentrates at adherens junctions near the cadherin–catenin complex, and activates the Hippo cascade, causing cells to exit the cell cycle.

When *NF2* is lost, or Hippo signaling is attenuated, however, YAP/TAZ are dephosphorylated, translocate to the nucleus and, together with TEAD1–4, initiate a transcriptional program of proliferation and survival. In parallel, YAP/TAZ shift Schwann cells toward a dedifferentiated, stem-like state. This transcriptional output has recently been shown to be not merely associated with resistance but required to sustain it. Two MPNST lines rendered resistant by approximately three months of continuous passage in selumetinib remained cross-resistant to fifteen further MEK inhibitors in a high-throughput screen—indicating that resistance had been fixed downstream of, rather than at, the MEK-binding site—and were re-sensitized by co-treatment with a TEAD inhibitor [45]. This identifies TEAD, for which several inhibitors have entered early clinical development, as a rational co-target in NF1-associated MPNST, and supplies the therapeutic corollary that this mechanism previously lacked. The report is, however, a short-format communication confined to two cell lines in vitro, without in vivo confirmation, and should be read as a strong hypothesis awaiting independent replication.

• *MNK/eIF4E signaling*

MNK1 and MNK2 (MAPK-interacting kinases) are serine/threonine kinases activated directly by several upstream inputs, including ERK1/2. Their only well-characterized protein substrate is eIF4E (eukaryotic translation initiation factor 4E), which is phosphorylated at Ser209 [34]. This event initiates the translation of oncogenic messenger RNAs. Even under MEK inhibition, this axis may remain active, sustaining proliferative activity and conferring resistance.

Despite blockade of ERK1/2, MNK activity is maintained by the following:

− p38α/β. These are two MAPK isoforms. MEK inhibitors switch off the RAF–ERK axis but do not affect p38. Moreover, MAPK inhibition and the accompanying cellular stress may themselves activate p38, which continues to feed MNK [35].
− Constitutive MNK2 activity. The isoforms differ: MNK1 is strongly dependent on stimulus-induced ERK/p38, whereas MNK2 possesses high basal, largely ERK-independent activity and sustains background phosphorylation of eIF4E even without an upstream signal [36].
− Residual ERK. MEK inhibitors rarely abolish phospho-ERK completely, and adaptive feedback loops (Section 3.5.1) partially restore ERK activity. This residual ERK is intermittently sufficient to reactivate MNK; the MNK axis is thus fueled by the same bypass mechanisms described above [37].

Phosphorylation of eIF4E at Ser209 by MNK kinases is critical for oncogenic transformation. It is, however, dispensable for normal development, which makes this signaling axis an attractive therapeutic target with a presumed low toxicity profile [38]. The evidence for this axis in nerve sheath tumors is direct rather than extrapolated: in NF1-mutant cancers, and specifically in MPNST, genetic and chemical suppression of MNK identified these kinases as therapeutic targets, and combined MNK and MEK inhibition induced tumor regression in vivo [39].

Type: **adaptive**—residual ERK together with p38/MNK2 sustains eIF4E-dependent translation immediately after MEK inhibition, constituting a rapid translational bypass. Model: NF1-mutant models (PN/MPNST); in vivo [39]. Evidence source: NF1-mutant PN and MPNST models; in vivo. Grade: E1/T2.

• *PI3K/AKT/mTOR signaling*

PI3K/AKT/mTOR is the principal parallel survival and growth pathway, which, in the setting of NF1 deficiency is activated concurrently with RAF/MEK/ERK: hyperactive RAS-GTP directly stimulates the catalytic subunit of PI3K, and the resulting PIP_3_ initiates the AKT → mTORC1 cascade that controls protein synthesis, metabolism, and apoptosis suppression. Because MEK inhibitors act only on the RAF–MEK–ERK axis, the PI3K/AKT/mTOR pathway remains unaffected and provides the tumor with an independent survival signal. Furthermore, ERK blockade releases negative feedback on RTKs, which further amplifies PI3K/AKT signaling; the pathway therefore acts not only as a constitutively active route but also as an adaptive escape mechanism. Persistent PI3K/AKT/mTOR signaling is recognized as one of the key drivers of malignancy in NF1-associated MPNST, and monotherapy—whether directed against MEK or against the pathway itself—has proved insufficient [47]. Until recently, the evidence for this mechanism within nerve sheath tumors was confined to MPNST. That gap has now been closed on the benign side: in immortalized patient-derived plexiform neurofibroma lines (ipNF95.11bC and ipNF05.5), 48-h exposure to four chemically and mechanistically distinct MEK inhibitors (selumetinib, binimetinib, CI-1040 and U0126) suppressed ERK phosphorylation but produced a reciprocal increase in the phosphorylation of AKT and of MEK itself, and the same pattern was reproduced in the MPNST line TRS462TY [48]. Two caveats must be stated. First, the readout is a phosphorylation signal and no rescue experiment was performed: co-activation of AKT has been shown to occur in PN, but not to be necessary for the survival of PN cells under MEK inhibition. Second, the finding is not reproduced in patients—in paired pre-treatment and on-treatment plexiform neurofibroma biopsies from the adult phase 2 study of selumetinib, phosphorylation of ERK1/2 fell by a median of 57–65% without any compensatory phosphorylation of AKT1/2/3 [17]. The mechanism therefore now carries direct preclinical support in PN, but the discrepancy between immortalized cell lines and patient tissue is unresolved and constitutes a specific, testable priority rather than a settled conclusion.

**Type: primary and adaptive.** The primary component is constitutive co-activation of PI3K/AKT/mTOR upon biallelic *NF1* loss: hyperactive RAS-GTP directly stimulates PI3K, so the pathway is active de novo, before treatment. The adaptive component is further amplification after MEK inhibition through release of ERK-dependent feedback on RTKs (see Section 3.5.2), which is rapid and reversible. Model: PN and MPNST cell lines (in vitro) [48], together with NF1-MPNST models in which loss of p53 shifts oncogenic signaling from the RAS/ERK axis to PI3K/AKT [43]; set against a negative pharmacodynamic signal in patient PN biopsies [17] and the negative clinical trial of selumetinib with sirolimus [46]. Evidence source: patient-derived PN and MPNST cell lines (adaptive AKT phosphorylation under four MEK inhibitors); paired on-treatment PN biopsies and the SARC031 trial, both negative. Grade: E2/T2.

• *CDK-dependent mechanisms*

CDK4/6 (cyclin-dependent kinases 4 and 6) are the executive node of the cell cycle downstream of MAPK: the RAS → RAF → MEK → ERK signal induces cyclin D, which activates CDK4/6, and these in turn phosphorylate retinoblastoma protein (RB1), releasing E2F transcription factors and initiating the G1 → S transition. Physiologically, it is through this axis that ERK executes its proliferative signal. Resistance develops when CDK4/6 becomes activated independently of the upstream signal, most often as a consequence of *CDKN2A/B* loss (the gene encoding p16^INK4a^, the physiological inhibitor of CDK4/6). Consequently, when a MEK inhibitor suppresses ERK and the supply of cyclin D, the cell cycle continues because the brake has been released at the level of the kinase itself. CDK4/6 thus confers resistance not by reactivating MAPK but by rendering cell cycle progression ERK-independent [33].

It should be emphasized that this mechanism applies predominantly to MPNST, owing to *CDKN2A* deletion. In benign PN, the gene is generally intact, which is consistent with the clinical efficacy of selumetinib in PN. CDK4/6-driven proliferation should therefore be regarded as a resistance mechanism principally in the context of MPNST [33].

**Type: primary**, since *CDKN2A* deletion is already present in MPNST. Model: MPNST in vitro and in vivo [33]. Evidence source: MPNST; in vitro and in vivo, with a CDK4/6–MEK inhibitor combination. Grade: E1/T1.

#### 3.5.3. Epigenetic and Transcriptional Rewiring

In this category, resistance is generated at the level of gene expression control rather than at the level of signaling cascade architecture.

• *Loss of the PRC2 complex through inactivation of its subunits (SUZ12, EED)*

PRC2 (Polycomb repressive complex 2) is a protein complex that represses genes epigenetically—that is, without altering the DNA sequence, by depositing a chemical mark on histones. This mark is H3K27me3, the trimethylation (me3) of lysine at position 27 (K27) of histone H3. Chromatin at such marked loci is tightly compacted, which prevents gene transcription.

The complex comprises four principal components:

− EZH2/EZH1 (Enhancer of Zeste Homolog 2/1)—the enzyme that deposits the H3K27me3 mark;
− EED (Embryonic Ectoderm Development)—participates in reading the mark and stimulates EZH2 to deposit further marks, generating positive feedback through which gene repression spreads along chromatin;
− SUZ12 (Suppressor of Zeste 12)—provides the structural scaffold of the complex, without which it cannot function;
− RBBP4/7 (Retinoblastoma-Binding Protein 4/7)—anchors the PRC2 complex to chromatin.

As described in Section 3.2, PRC2 loss is a frequent event in MPNST. The mechanisms outlined below circumvent the principal node targeted by MEK inhibitors and drive tumor proliferation as a consequence of complex loss.

According to De Raedt et al. (Nature, 2014), RAS signaling is executed through the “opening” of genes bearing the activating mark H3K27ac (acetylation of lysine 27). While PRC2 is functional, a balance is maintained between methylation (repression) and acetylation (activation) of lysine 27. Upon its loss, the equilibrium shifts toward activation. BRD4 (bromodomain-containing protein 4) is recruited to such acetylated loci; this protein belongs to the BET family, reads H3K27ac marks, and stabilizes the p300/CBP complex responsible for acetylation [40].

This mechanism clearly bypasses the drug-inhibited node (MEK) at a point far downstream of ERK—at the level of transcription. It should be noted that the two components of this account rest on different amounts of evidence. The frequency of PRC2 inactivation in MPNST has been established independently in several cohorts [15] and its therapeutic corollary has been tested in combination experiments in MPNST models [41]; the specific mechanistic model—loss of PRC2 shifting the H3K27me3/H3K27ac balance and recruiting BRD4 to amplify RAS-driven transcription—derives principally from a single study [40] and has not been independently replicated in nerve sheath tumors.

**Type: primary**, because PRC2 inactivation is an early, defining event of malignant transformation in MPNST that exists before treatment and shifts transcription downstream of ERK. Model: MPNST (mouse); in vivo [40,41]. Evidence source: MPNST (murine models and human tumor material); in vivo. Grade: E1/T3.

**Figure 7:**
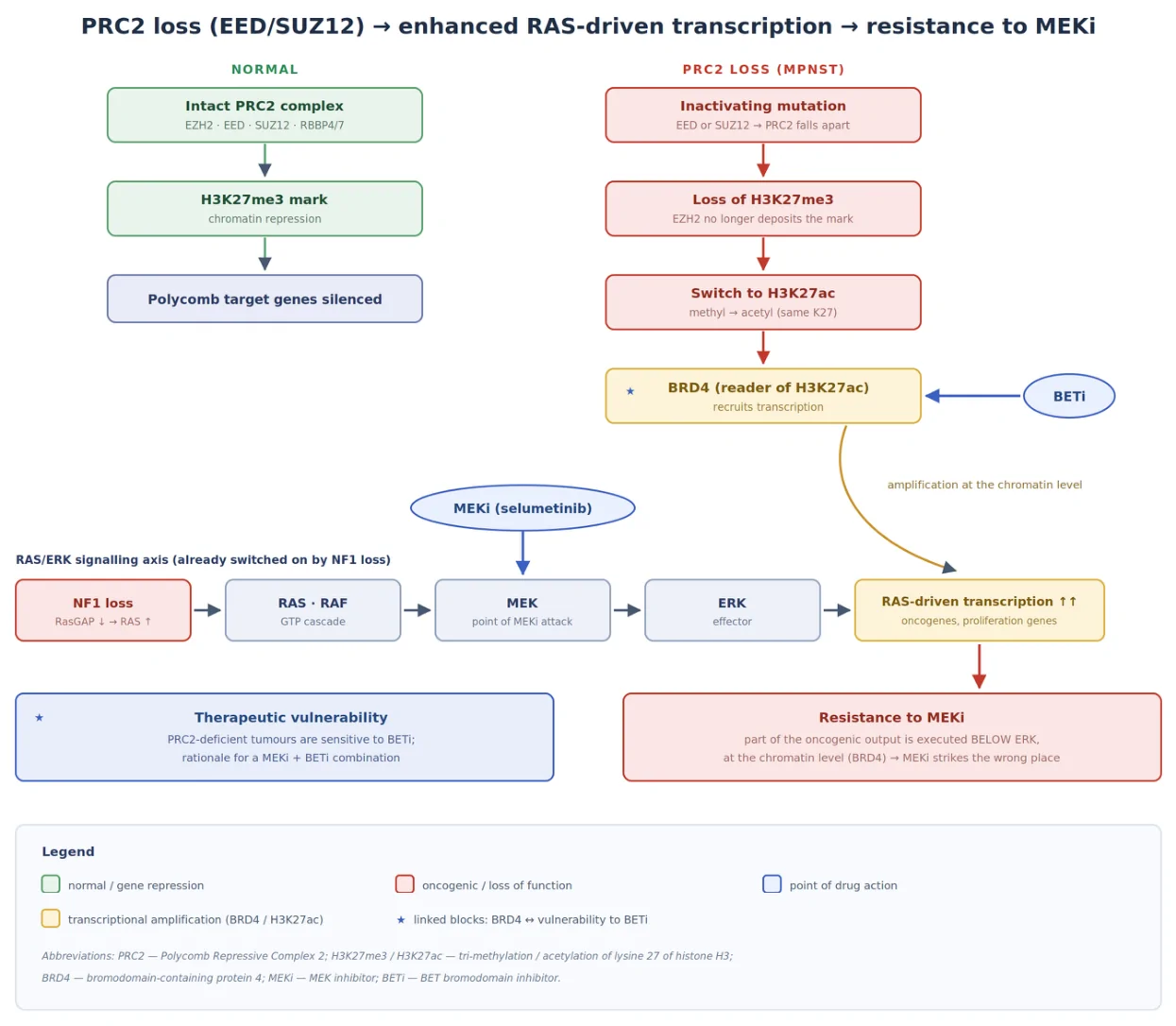
Scheme of epigenetic and transcriptional rewiring following PRC2 loss.

An alternative mechanism of RAS signal amplification also linked to PRC2—specifically to SUZ12 loss—is reactivation of the *ADCY1* (adenylate cyclase 1) gene. Li W. et al. (Frontiers in Oncology, 2021) identified *ADCY1* as a key gene through protein–protein interaction analysis combined with survival analysis in patients with MPNST, after which the mechanism was validated in plexiform neurofibroma cells. The expressed adenylate cyclase subsequently catalyzes the formation of cAMP, which, as a universal intracellular second messenger, activates PKA (protein kinase A), which in turn activates RAP1 (Ras-related protein 1), a small GTP-binding protein of the Ras family. RAP1 binds and activates BRAF (in contrast to RAF-1/CRAF, which it tends to inhibit) [42]. It should be noted, however, that *ADCY1* reactivation is not an autonomous mechanism but rather a factor that increases the baseline dependence of tumor progression on the MAPK cascade (Figure 7).

• *BET-mediated kinome reprogramming*

Kinome reprogramming is the reorganization of the entire complement of active cellular kinases in response to the inhibitor itself. This mechanism is not a genetic mutation but a rapid and reversible transcriptional response. Tumor cells “rewire” their signaling to bypass the blocked node. In the present context, kinome reprogramming corresponds to transcriptional induction of new RTKs following release of ERK-dependent feedback.

BET proteins are a family of proteins that recognize acetylated lysine residues on histones, allowing them to influence chromatin accessibility, recruit transcription factors, and drive expression of adaptation genes. It is these proteins that mediate transcriptional rewiring.

Section 3.5.2 described the problem of heterogeneous reactivation of multiple RTKs under MEK inhibition, which makes individual inhibition of each receptor impractical. Whereas SHOC2 is the link between RTKs and RAS at the biochemical level, BET proteins represent the transcriptional hub that switches on all RTKs [68,69].

**Type: adaptive**—a rapid, reversible, gene-independent transcriptional response to the inhibitor itself (release of ERK feedback → RTK induction). Model: triple-negative breast cancer (cell lines plus genetically engineered mouse models); in vitro and in vivo; direct transferability to PN/MPNST has not been demonstrated [68]. Evidence source: triple-negative breast cancer; not demonstrated in nerve sheath tumors. Grade: E3/T1.

FOSL1 (FOS-like antigen 1), reported by Kurimchak et al. in NF1-deficient ovarian cancer, merits separate mention. FOSL1 is a transcription factor whose protein stability is maintained by ERK-mediated phosphorylation; the protein therefore remains stable only while the MAPK cascade is active. Its function is to restrain RTK expression. Under MEK inhibition of the MAPK cascade, FOSL1 is destabilized and RTK genes are correspondingly de-repressed. BRD2 and BRD4 (BET proteins) mediate this RTK induction; synthetic enhancement screens in that study identified BRD2 and BRD4 as obligatory mediators of the MEK inhibitor-induced RTK signature [69].

Type: **primary.** The BET-dependent state is present *before* therapy. Model: NF1-deficient ovarian cancer; transferability to MPNST has not been proven; in vitro [69]. Evidence source: NF1-deficient ovarian cancer; in vitro; not demonstrated in nerve sheath tumors. Grade: E3/T1.

• *Loss of p53 function*

Under physiological conditions, the cytostatic and pro-apoptotic response to MEK inhibition is largely executed through p53-dependent programs—cell cycle arrest via p21 and induction of apoptosis. Inactivation of p53 raises the threshold for cell death, so that a fraction of cells survives therapeutic pressure as drug-tolerant persisters. It is against this background of prolonged survival that the escape mechanisms proper have time to develop—adaptive kinome reprogramming through release of ERK-dependent feedback, and partial loss of differentiation toward a less MAPK-dependent state. These processes can occur independently of p53 status, but they are amplified when p53 is dysfunctional.

According to Grit et al., using genetically characterized murine NF1-MPNST models (including an isogenic CRISPR knockout of p53) and a panel of human cell lines, p53 loss confers resistance to MET and MEK inhibition despite preserved suppression of phospho-ERK: the oncogenic signal shifts from the RAS/ERK axis to PI3K/AKT [43].

**Type: primary**—p53-deficient cells are intrinsically resistant to MET/MEK inhibition despite preserved ERK suppression. The mechanism is genetically determined (*TP53* loss). Model: NF1-associated MPNST (murine models, isogenic CRISPR p53 knockout and human cell lines); in vitro/in vivo [43]. Evidence source: NF1-associated MPNST (isogenic CRISPR *TP53* knockout, murine and human cell lines); in vitro and in vivo. Grade: E1/T3.

#### 3.5.4. Cell Survival Programs

In this category, the cell—while restoring signaling—avoids death, blocks apoptosis or switches to catabolic survival (protective autophagy). These are biologically plausible and potentially targetable survival programs which, for the most part, have not yet been characterized in nerve sheath tumors and represent an area of interest for further research.

• *Survivin and anti-apoptotic programs*

Survivin (BIRC5) is overexpressed in pediatric MPNST and correlates with higher tumor grade and worse survival [76].

A mechanistically relevant link was demonstrated by Sumi et al. (2018): in RB1-competent KRAS-mutant cells, trametinib suppresses survivin indirectly—through p21 induction and RB1 dephosphorylation—whereas in RB1-deficient cells suppression was only transient, resistance developed rapidly, and the resistant cells became survivin-dependent [77]. The key inference is that suppression of survivin by MEK inhibitors is conditional and requires dephosphorylated RB1. This makes extrapolation to MPNST justified, since malignant transformation of plexiform neurofibromas is accompanied by inactivation (hyperphosphorylation) of the RB pathway resulting from loss of p16, which is capable of promoting RB1 dephosphorylation. This most often arises from homozygous loss of *CDKN2A*/p16^INK4A^, characteristic of ANNUBP [6,84]. However, p16^INK4A^ and p21 are not interchangeable: p16 is a specific inhibitor of CDK4/6, whereas MEK inhibitor-induced p21 acts predominantly on other CDK complexes. Homozygous loss of *CDKN2A*/p16^INK4A^ therefore disinhibits CDK4/6 and sustains constitutive hyperphosphorylation of RB1, which p21 induction may be insufficient to reverse. For this reason, in p16-deficient tumors the p21-dependent arm of the mechanism described by Sumi et al. fails, and MEK inhibitors lose the ability to switch off survivin—which converts survivin persistence from a prognostic marker into a candidate mechanism of intrinsic resistance. CDK1, selectively elevated in MPNST and associated with adverse prognosis [78], plays only an ancillary role here, as a mitotic marker that stabilizes survivin through phosphorylation at Thr34 [79].

Direct evidence for a contribution of survivin to MEK inhibitor resistance in MPNST is lacking, and the mechanisms verified in NF1 models relate to RAS reactivation [27,31,47]. The hypothesis therefore requires targeted validation—assessment of *BIRC5* dynamics and of p21–RB axis status under MEK inhibition in models stratified by *CDKN2A*/*RB1*, together with a functional test of whether survivin suppression (YM155) restores MEK inhibitor sensitivity in RB-deficient tumors.

**Type: primary (candidate mechanism).** Survivin (BIRC5) overexpression in MPNST results from increased copy number at the 17q locus, is present before treatment, and is associated with higher grade and worse survival—that is, it represents a pre-existing anti-apoptotic state. A direct link with resistance to MEK inhibition specifically has not been demonstrated [77], so the mechanism is primary in nature but remains a candidate with respect to MEK inhibitors. Model: prognostic MPNST data (patient specimens); in vitro. Evidence source: MPNST (prognostic data from patient specimens); the mechanistic link with MEK inhibition has been shown only in RB1-deficient KRAS-mutant lung adenocarcinoma. Grade: E3/T3.

• *Protective autophagy (under SHP2 inhibition)*

The concept of cytoprotective autophagy as an adaptive mechanism of resistance to blockade of the RAS–RAF–MEK–ERK cascade has been characterized in RAS-driven tumor models: MEK1/2 inhibition activates the LKB1/AMPK/ULK1 axis and increases autophagic flux, protecting cells from the cytotoxic effect of the blockade, while concurrent suppression of KRAS/ERK inhibits glycolysis and mitochondrial metabolism, increasing dependence on autophagy. Combining a MEK inhibitor with an autophagy inhibitor acts synergistically in vitro and in patient-derived xenografts of melanoma, pancreatic and colorectal cancer [50,51]. Clinical evaluation in BRAF-V600-mutant melanoma (BAMM, phase I/II: hydroxychloroquine plus dabrafenib and trametinib) yielded a high response rate (85%, with 41% complete responses) and a median overall survival of 26.5 months, but the prespecified primary endpoint (one-year progression-free survival >53%) was not met—the observed value was 48.2% (95% CI 31.0–65.5%). The result is more accurately interpreted as falling short of the prespecified threshold while providing an encouraging signal, which served as the rationale for a randomized trial [49].

Direct data in peripheral nerve sheath tumors exist but were not obtained with MEK inhibitors. In MPNST models (genetically engineered and orthotopic patient-derived xenografts), MEK inhibition showed weak antitumour activity, whereas upstream RAS inhibition through SHP2 suppressed tumor growth; acquired resistance developed over time, and kinome analysis of the resistant tumors revealed enrichment for activated kinases of the autophagy pathway, with the addition of hydroxychloroquine to the SHP2 inhibitor producing a durable response [31].

Thus, in MPNST autophagy is documented specifically as a mechanism of acquired resistance, but (i) to SHP2 rather than MEK inhibition, and (ii) in a context in which single-agent MEK inhibition is intrinsically of limited efficacy—which constrains direct transfer of the melanoma–pancreatic paradigm of “MEK inhibition → protective autophagy” to this entity.

The role of MEK inhibitor-induced protective autophagy in MPNST is currently unproven and is inferred by analogy with melanoma and pancreatic models. Confirmation of this hypothesis requires dedicated studies in relevant nerve sheath tumor models, with genetic confirmation of autophagy dependence (knockout or knockdown of *ATG5*/*ATG7*, *ULK1*) in addition to pharmacological blockade, followed by clinical evaluation of MEK inhibitor plus autophagy inhibitor combinations.

**Type: adaptive or acquired (context-dependent; not established for MEK inhibition in MPNST).** In the general RAS paradigm (melanoma, pancreas, colon), cytoprotective autophagy is an adaptive response: MEK inhibition rapidly and reversibly activates the LKB1/AMPK/ULK1 axis and increases autophagic flux. In the only direct MPNST data, it is documented as acquired resistance—developing under prolonged exposure, but to SHP2 rather than MEK inhibition, and against a background of intrinsically limited single-agent MEK inhibitor efficacy. MEK inhibitor-induced protective autophagy in MPNST remains unproven. Model: melanoma/pancreatic/colorectal patient-derived xenografts plus BRAF-mutant melanoma (BAMM) for the paradigm; MPNST models for SHP2 inhibition. Evidence source: MPNST, but under SHP2 rather than MEK inhibition (genetically engineered and orthotopic patient-derived xenografts); MEK inhibitor-induced protective autophagy has been shown only in melanoma, pancreatic and colorectal models. Grade: E2/T2.

• *The BH3 axis: BIM–MCL-1–BCL-2*

Under physiological conditions, active ERK maintains the balance in favor of survival from two directions. First, ERK phosphorylates the pro-apoptotic BH3 protein BIM (BCL2L11) at Ser69 and, in parallel, phosphorylates FOXO3A, which suppresses *BIM* transcription. Second, ERK phosphorylates the anti-apoptotic protein MCL-1, slowing its turnover. Upon MEK inhibition, both arms are reversed: BIM accumulates and is stabilized. Accumulated BIM does not, however, trigger apoptosis if it is bound and neutralized by anti-apoptotic proteins—MCL-1, BCL-2 and, above all, BCL-xL. It is the sequestration of BIM by anti-apoptotic proteins that determines whether cell death occurs or the cell is merely stabilized without further growth [52,53].

According to Park et al. (2013), BCL-xL is elevated in MPNST tissue compared with plexiform neurofibromas, and in Schwann cells derived from MPNST, BCL-xL overexpression is directly attributable to NF1 deficiency through enhanced Ras/MAPK signaling and confers resistance to apoptosis induction [54]. The anti-apoptotic tone of MPNST is additionally sustained post-translationally: the Sec6/8 (exocyst) complex regulates BCL-2 and MCL-1 levels in MPNST cells [55].

**Type: adaptive and primary.** The primary component is the intrinsically elevated BCL-xL level in NF1-deficient cells (the threshold state exists before therapy); the adaptive component is the rapid accumulation of BIM after MEK inhibition which, without co-inhibition of the BCL-2 family, is not converted into cell death. **Model:** direct expression data from NF1-MPNST (in vitro and patient specimens). Evidence source: NF1-associated MPNST (cell lines and patient specimens); expression-level data. Grade: E2/T2.

#### 3.5.5. Tumor Microenvironment and Immunity

This category comprises resistance that arises outside the tumor cell, through the surrounding histological structures. It should be noted that direct studies demonstrating that modulation of the tumor microenvironment can restore MEK inhibitor sensitivity are currently insufficient; it cannot therefore be stated with confidence that the mechanisms described below directly affect the quality of MEK inhibitor therapy. Nevertheless, this section may be valuable for further experimental investigation of MEK inhibitor resistance.

• *CD163^+^ tumor-associated macrophages and myeloid immune evasion*

As NF1-associated nerve sheath tumors undergo malignant transformation, their microenvironment becomes markedly enriched in immune cells, with the myeloid rather than the lymphoid compartment predominating—above all CD163^+^ tumor-associated macrophages (TAM). In the largest immunophenotypic study of human material, these macrophages in MPNST show high PD-L1 expression, neighboring T cells appear exhausted (high PD-1), and the proportion of CD163^+^ myeloid cells is inversely associated with progression-free survival [56]. The tumor becomes immunosuppressive, with macrophages rather than T cells setting the tone. The biology of the benign stage is consistent with this: already in plexiform neurofibroma, macrophages are recruited early and in large numbers, supporting tumor growth through the MIF–CD74/NF-κB and STAT3 axes, and their pharmacological suppression reduces tumor burden in model systems [57,58]. An indirect link with therapy is provided by the observation that MEK inhibitors (in combination with a SOS1 inhibitor) alter macrophage morphology and activation profile, indicating that the drug reaches the myeloid compartment and not only the tumor cell [28].

Future studies should determine whether macrophage depletion (for example, CSF1R blockade) influences the response to MEK inhibitors in MPNST models, and should characterize TAM specifically in PN rather than extrapolating from the malignant stage.

**Type: primary.** Immunosuppressive myeloid infiltration (CD163^+^ TAM with high PD-L1 expression) is a pre-existing property of the microenvironment that increases with malignant progression of NF1-associated tumors; it exists before and independently of MEK inhibitor exposure, which corresponds to the definition of primary (pre-existing) resistance operating outside the tumor cell. A causal relationship with the response to MEK inhibition has not been demonstrated—the association is at present prognostic and requires validation (for example, TAM depletion by CSF1R blockade in MPNST models). Model: MPNST—immunophenotyping of surgical material; PN—murine models; clinical specimens and in vivo data. Evidence source: MPNST (immunophenotyping of surgical specimens; prognostic association) and PN (murine models); no causal test under MEK inhibition. Grade: E2/T2.

• *FOXM1 as a transcriptional mediator of acquired resistance and immune evasion*

FOXM1 is an oncogenic transcription factor that activates genes of cell division and DNA repair and protects the cell from apoptosis. Interest in FOXM1 arose from an observation in MPNST models: the combination of CDK4/6 and MEK inhibitors initially induces tumor regression and even confers sensitivity to anti-PD-L1 therapy, but over time the tumor escapes—its microenvironment becomes immunosuppressive, MHC-II-low macrophages accumulate, and the tumor cells themselves upregulate PD-L1 [33]. FOXM1, which is closely linked to PD-L1 regulation, is proposed by the authors as a component of escape: it simultaneously restarts proliferation around the blocked kinases and dampens antitumour immunity [62].

For MPNST, however, the role of FOXM1 remains hypothetical: it has not yet been shown that FOXM1 suppression restores therapeutic sensitivity, and the link with immune evasion is largely transposed from other tumors. Moreover, the observation concerns dual CDK4/6 plus MEK targeting rather than single-agent MEK inhibition, so the conclusion cannot be transferred directly to clinical use of selumetinib. To establish the true relevance of this mechanism, FOXM1 must be tested functionally in paired sensitive and resistant MPNST models.

**Type: acquired** (a mediator of escape from CDK4/6 plus MEK inhibition; not demonstrated under single-agent MEK inhibition). Model: MPNST, preclinical; the link with PD-L1 is partly extrapolated; in vitro/in vivo. Evidence source: MPNST, preclinical; demonstrated under combined CDK4/6–MEK inhibition, not under single-agent MEK inhibition. Grade: E2/T3.

• *Stroma, extracellular matrix and paracrine protection from MEK inhibitors*

Plexiform neurofibroma consists predominantly of stroma: fibroblasts are the most abundant population of the microenvironment, and the tumor Schwann cells are embedded in a dense desmoplastic matrix. This environment appears to reduce MEK inhibitor efficacy. In three-dimensional co-cultures of human NF1-deficient Schwann cell-derived tumor cells with patient fibroblasts, tumor structures became markedly more resistant to selumetinib and mirdametinib than in monoculture; the fibroblast secretome, acting through vesicles (including via conditioned medium, without direct cell contact), increased P-glycoprotein (P-gp) expression in tumor cells, and pharmacological P-gp blockade partially restored sensitivity [63]. The same secretome stimulates the growth and invasion of Schwann cell-derived tumor cells [64].

The contribution of the extracellular matrix (ECM) has been demonstrated separately: loss of neurofibromin sensitizes cells to signals from a “PN-like” ECM through FAK hyperactivation (with phosphorylation of Src, ERK and AKT), and whereas selumetinib alone suppresses colony growth only partially, its combination with a FAK inhibitor (defactinib) blocks growth completely [65]. The mechanical properties of that matrix, and not only its biochemical composition, have since been shown to matter independently: in three-dimensional cultures of immortalized patient-derived PN cells, a stiff hydrogel (7 kPa) as compared with a soft one (1.5 kPa) promoted spread morphology and growth, reduced lysyl oxidase expression, increased P-glycoprotein expression and was associated with reduced sensitivity to selumetinib [66]. Because postsurgical remodeling of residual tumor produces precisely this kind of localized stiffening, the observation raises the clinically relevant possibility that an incompletely resected residuum becomes less, rather than more, tractable to subsequent MEK inhibition. Prolonged MEK inhibition has itself been shown to increase extracellular matrix proteolysis in three-dimensional PN structures [48], so drug and matrix appear to act on one another reciprocally. Neither observation has been tested in patients.

**Type: primary.** Protection conferred by stroma and ECM is a pre-existing tissue property that is not drug-induced: the dense matrix and paracrine fibroblast signals increase P-glycoprotein expression (efflux of selumetinib), while loss of neurofibromin sensitizes cells to a “PN-like” ECM through FAK hyperactivation (with phosphorylation of Src, ERK and AKT). These factors are present before therapy and reduce the effect of MEK inhibitors even before resistant clones are selected, which corresponds to the definition of primary resistance. Model: PN—three-dimensional co-cultures and hydrogels of defined stiffness; in vitro (3D). Evidence source: PN (three-dimensional cultures and murine models) and MPNST; in vitro and in vivo. Grade: E2/T3.

• *Immunological consequences of MEK inhibition*

MEK blockade alters not only the tumor cell but also the immune environment, and the effect may be bidirectional.

There is a negative effect that may reasonably be regarded as a resistance mechanism. Because T-cell receptor signaling physiologically proceeds through the MEK/ERK cascade, MEK inhibition may dampen activation of T cells themselves. This has been confirmed functionally: MEK inhibition contracts dominant intratumoural T-cell clones, and only the addition of a type II RAF inhibitor reverses this effect and restores clonal expansion [70].

Conversely, MAPK inhibition also enhances the production of chemokines that attract cytotoxic T cells and increases tumor susceptibility to checkpoint blockade; mechanistically this has been shown outside NF1: combining a MEK inhibitor with chemotherapy induces CXCL10 and CD8^+^ recruitment into the tumor [71], while adding a STAT3 inhibitor to a MEK inhibitor reprograms macrophages from M2 to M1 and remodels the stroma [72]. This logic is reproduced in NF1 models, but always within combination regimens: dual CDK4/6 plus MEK blockade in MPNST elicits an unusual plasma cell infiltrate and an increase in cytotoxic T cells, sensitizing the tumor to anti-PD-L1 [33], while combining selumetinib with an RXR agonist reduces phospho-ERK and the number of tumor-supporting macrophages and increases activated CD8^+^ T cells in a syngeneic MPNST model [73].

Two caveats apply. First, almost all “pro-immune” data in NF1 derive from combination regimens rather than single-agent MEK inhibition. Second, the mechanistic depth (CXCL10, M2→M1 polarization, T-cell clonality) was obtained in melanoma, lung and pancreatic cancer; in NF1 it rests on two preclinical studies and requires direct validation in PN and MPNST models.

**Type: adaptive (for the resistance component).** The pro-resistance effect is direct, rapid and reversible suppression of MEK/ERK-dependent T-cell receptor signaling, weakening the antitumour T-cell response during drug exposure; it is gene-independent and resolves upon withdrawal, which corresponds to the definition of adaptive resistance—here operating in the immune compartment. The opposing pro-immune effect (chemokine induction, CD8^+^ recruitment, sensitization to checkpoint blockade) is not a resistance mechanism and is not included in the taxonomy of resistance types; in NF1 it has been demonstrated only for combinations, and mechanistically only outside NF1. Model: mechanistic data outside NF1 (melanoma, lung, pancreas); in NF1, combinations only; in vivo and extrapolation. Evidence source: predominantly non-NF1 models; in NF1 models demonstrated only within combination regimens. Grade: E3/T2.

#### 3.5.6. Intratumoural Heterogeneity

Intratumoural heterogeneity is considered here as a possible source of incomplete response to MEK inhibitors. As in the two preceding sections, direct confirmation in PN and MPNST under MEK inhibition was not identified: the evidence base rests on transcriptional surrogates (in silico) and on analogy with NF1-mutant glioblastoma. The mechanism is therefore presented as an extrapolation with potential for further investigation rather than as an established route.

• *Single-cell differential sensitivity of Schwann cells*

Single-cell sequencing technologies (scRNA-seq/snRNA-seq) have established that the tumor is not a homogeneous mass of identical cells. It contains several Schwann cell populations that may respond differently to MEK inhibition. Some cease dividing and die, whereas others—predominantly dedifferentiated cells—survive without responding to the drug. Selective sensitivity of tumor cell subsets may explain the partial response to MEK inhibitors: the tumor shrinks but does not disappear completely.

In an in silico analysis of seven plexiform neurofibromas using a drug sensitivity signature (BeyondCell), statistically significant differences in the computed selumetinib response profile were identified between tumors [67].

It should be noted, however, that computed sensitivity estimates indicate a dedifferentiated, slowly dividing cell state (resistant to therapy in general) rather than specific resistance to MEK inhibition, which must be taken into account when interpreting this mechanism.

Integration of bulk and single-cell profiling has further shown that loss of the differentiated Schwann cell phenotype, driven by *NF2* inactivation and PAK activation (Section 3.5.2), is associated with resistance to selumetinib, and that the dedifferentiated subpopulation persists under drug exposure in vivo [26].

**Type of resistance:** primary (pre-existing heterogeneity). **Tumor type:** predominantly PN (not studied in MPNST). **Level of evidence:** low—transcriptional surrogates (scRNA-seq/snRNA-seq, in silico) and xenograft models, without direct functional verification or clinical correlation. Evidence source: PN (scRNA-seq/snRNA-seq and in silico drug-response signatures); not studied in MPNST. Grade: E2/T3.

• *Mesenchymal-like (MES-like) and non-mesenchymal subpopulations*

A separate axis of intratumoural heterogeneity relevant to MEK inhibitor response is the contrast between mesenchymal-like (MES-like) and non-mesenchymal (non-MES) tumor states, a distinction developed and validated for glioblastoma [80]. In somatically NF1-mutant glioblastoma, MES-like cells are enriched for a transcriptional signature of MEK/ERK activation relative to the non-MES compartment, and MEK inhibition selectively depleted them, while surviving MES-like cells reactivated Ras signaling. The functional relevance of the cascade was confirmed by CRISPRi, and repression of SHOC2 sensitized tumors to the drug [81]—the same node identified independently in NF1-deficient nerve sheath tumors (Section 3.5.1).

Direct evidence of differential MEK inhibitor sensitivity along the MES/non-MES axis was obtained in NF1-mutant glioblastoma, and its transfer to PN/MPNST is extrapolated. The MES/NPC/OPC/AC framework itself is validated for glioma and has not been applied directly to Schwann cell tumors. In MPNST, a mesenchymal-like subpopulation has been described, but its response specifically to MEK inhibition has not been tested; the key evidence (depletion of MES-like cells under selumetinib) was obtained in murine models [82]. For plexiform neurofibromas, no data on the MES/non-MES axis are available.

Model: NF1-mutant glioblastoma; by analogy, MPNST. Type of resistance: primary, with adaptive Ras reactivation in surviving MES-like cells. Evidence source: NF1-mutant glioblastoma; extrapolated to MPNST. Grade: E3/T3.

## 4. Conclusions

On the basis of analysis, every mechanism was graded along two independent axes.

The first axis is the strength of evidence that the mechanism confers resistance to MEK inhibition in NF1-associated PN or MPNST. E1: demonstrated in PN or MPNST with a functional perturbation experiment—the mechanism is engaged under MEK inhibition, and its blockade, silencing, or genetic removal restores sensitivity, or the corresponding combination is effective in vivo. E2: demonstrated in PN or MPNST, but descriptively or associatively only (expression, phosphoproteomic, transcriptomic, or prognostic data without a rescue experiment), or demonstrated functionally but under a different inhibitor class. E3: extrapolated—functional evidence exists only in NF1-wild-type RAS- or RAF-driven tumors of another lineage, or in another NF1-deficient tumor type, with no data in nerve sheath tumors.

The second axis is therapeutic tractability. T1: a direct pharmacological target exists, agents are in clinical use or in clinical trials, and combination data in PN or MPNST models are available. T2: a direct target and clinically available agents exist, but combination data in PN or MPNST are absent, incomplete, or clinically negative. T3: no direct pharmacological target; the mechanism can be addressed only indirectly, through synthetic lethality, vertical inhibition of the cascade or modulation of the lineage state.

This two-axis grading was constructed for the present review and is not an established scale. It follows the logic of clinical actionability frameworks—ESCAT [94], in which tier III likewise marks evidence obtained in a different tumor type, and OncoKB [95], which grades biomarkers of resistance alongside biomarkers of sensitivity—but neither applies directly here: both rank alteration–drug pairs on clinical evidence, whereas the data on resistance to MEK inhibition in PN and MPNST are almost entirely preclinical and concern mechanisms rather than driver alterations.

The two axes are deliberately orthogonal, and the grades are assigned independently. A mechanism may be firmly established and untargetable, or targetable and poorly substantiated; both situations are informative. The grades of all mechanisms are given in Table 1, together with the entity and model that constitute the evidence source in each case.

Two examples show why the axes must be kept apart. Loss of p53 function is graded E1/T3: it is the best-documented determinant of resistance in NF1-associated MPNST—isogenic *TP53*-knockout cells survive MET/MEK inhibition despite full suppression of ERK phosphorylation—yet no agent restores p53 function, so the mechanism carries no direct therapeutic action and can be approached only through synthetic lethality or lineage-state modulation. BET-mediated kinome reprogramming is graded E3/T1: BET inhibitors are in clinical development, and the combination is straightforward to test, but the reprogramming itself has been shown in triple-negative breast and NF1-deficient ovarian cancer and not in nerve sheath tumors. Under the previous scheme, both mechanisms occupied the same tier, which said nothing about how different the underlying evidence is.

Co-activation of PI3K/AKT/mTOR makes the same point from the clinical side and is graded E2/T2. Adaptive AKT phosphorylation under four different MEK inhibitors has been shown directly in patient-derived PN and MPNST cell lines, but no experiment has yet demonstrated that blocking it restores MEK inhibitor sensitivity in these models, and in paired on-treatment biopsies from adults with PN, AKT phosphorylation did not rise while ERK phosphorylation fell by a median of 57–65% [17,48]. The negative result of SARC031 (selumetinib plus sirolimus in MPNST) therefore constrains the tractability axis and not the evidence axis: a negative combination trial shows that one schedule of one drug pair failed in one setting, not that the pathway is uninvolved in resistance. For this reason, no mechanism is excluded from Table 1 on the basis of a negative trial.

The key limitation of the field remains the scarcity of clinical data on resistance in patients with PN and MPNST receiving MEK inhibitors. Priorities are prospective molecular monitoring of resistance in patients, clinical evaluation of rational combinations, and the identification of biomarkers that stratify tumors according to the dominant escape mechanism. Two recent developments make these priorities more tractable than they were. Patient-derived orthotopic xenograft platforms have now been used prospectively to inform molecular tumor board decisions in MPNST, providing a route by which mechanism-stratified combinations can be tested against an individual tumor before commitment to a trial [96]. In parallel, unbiased screening across *NF1-deficient models is generating combination candidates that pathway reasoning alone would not have predicted* [29]. A systematic account of the combinations now entering the clinic against these nodes—SHP2, SOS1 and CDK4/6 inhibitors partnered with MEK inhibition—lies outside the scope of the present work, which concerns the mechanisms of escape themselves; that landscape is the subject of a separate review in preparation by the present authors.

## Figures and Tables

**Table 1:** Mechanisms of resistance to MEK inhibitors in NF1-associated plexiform neurofibroma and MPNST, graded independently for strength of evidence in these entities (E1–E3) and for therapeutic tractability (T1–T3). The evidence source states the tumor entity and the model in which the mechanism was demonstrated. Grading criteria are defined in the text above. Mechanisms are ordered by evidence grade and then by tractability.

Mechanism (Section)	Evidence Source: Entity and Model	E	T	Ref.
Release of ERK-dependent negative feedback with RAF dimerization (SHOC2/PP1)—3.5.1	NF1-deficient MPNST; murine models and cell lines; RAF dimer inhibitor combination in vivo	E1	T1	[23,24,25,26]
Convergence of bypass inputs on SHP2 (PTPN11) → SOS1—3.5.1	NF1-deficient MPNST; cell lines and murine models; SHP2 inhibitor plus MEK inhibitor in vivo	E1	T1	[27,28,29]
Parallel reactivation through RTKs (HGF/MET, PDGFRβ)—3.5.2	MPNST; resistant cell lines with HGF/MET silencing and rescue; murine models	E1	T1	[24,30,31,32]
Cooperation with the cell cycle: CDK4/6 under CDKN2A/B loss—3.5.2	MPNST; in vitro and in vivo, with a CDK4/6–MEK inhibitor combination	E1	T1	[33]
MNK/eIF4E translational bypass—3.5.2	NF1-mutant PN and MPNST models; in vivo	E1	T2	[34,35,36,37,38,39]
PRC2 loss (SUZ12/EED) with amplified RAS-driven transcription (ADCY1)—3.5.3	MPNST; murine models and human tumor material	E1	T3	[40,41,42]
Loss of p53 function—3.5.3	NF1-associated MPNST; isogenic CRISPR TP53 knockout, murine and human cell lines	E1	T3	[43]
Integrin/adhesion axis LAMA4/ITGB1/FAK/SRC—3.5.2	Resistant MPNST cell lines; in vitro only	E2	T2	[44]
NF2 loss, PAK activation and Schwann cell dedifferentiation (Hippo–YAP/TAZ–TEAD)—3.5.2	MPNST and PN; TEAD-inhibitor resensitization in two MPNST lines in vitro	E2	T2	[26,45]
Co-activation of PI3K/AKT/mTOR—3.5.2	Patient-derived PN and MPNST cell lines; paired on-treatment PN biopsies and SARC031, both negative	E2	T2	[17,43,46,47,48]
Protective autophagy—3.5.4	MPNST, but under SHP2 rather than MEK inhibition; MEK inhibitor-induced autophagy only in melanoma, pancreatic and colorectal models	E2	T2	[31,49,50,51]
BH3 axis: BIM–MCL-1–BCL-2/BCL-xL—3.5.4	NF1-associated MPNST; cell lines and patient specimens; expression-level data	E2	T2	[52,53,54,55]
CD163^+^ tumor-associated macrophages and myeloid immune evasion (PD-L1)—3.5.5	MPNST surgical specimens (prognostic association); PN murine models; no causal test under MEK inhibition	E2	T2	[56,57,58]
Loss of DUSP/SPRY-mediated restraint of ERK—3.5.1	NF1 peripheral nerve tissue atlas and MPNST cell lines; expression-level and pharmacodynamic data	E2	T3	[59,60,61]
FOXM1 as a transcriptional mediator of escape and immune evasion—3.5.5	MPNST, preclinical; under combined CDK4/6–MEK inhibition, not single-agent	E2	T3	[33,62]
Stroma, extracellular matrix stiffness, paracrine protection and P-glycoprotein—3.5.5	PN three-dimensional cultures and murine models; MPNST	E2	T3	[48,63,64,65,66]
Intratumoural heterogeneity and single-cell differential sensitivity—3.5.6	PN; scRNA-seq/snRNA-seq and in silico drug-response signatures; not studied in MPNST	E2	T3	[26,67]
BET-mediated kinome reprogramming—3.5.3	Triple-negative breast cancer;	E3	T1	[68]
FOSL1 destabilization → BRD2/BRD4-mediated RTK induction—3.5.3	NF1-deficient ovarian cancer;	E3	T1	[69]
Bidirectional immunological effect of MEK inhibition—3.5.5	Predominantly non-NF1 models; in NF1 models only within combination regimens	E3	T2	[33,70,71,72,73]
KRAS/NRAS amplification or mutation—3.5.1	NF1-wild-type melanoma and colorectal cell lines of non-nerve-sheath lineage; in vitro	E3	T3	[25,74]
P-body dissolution with increased RAS translation—3.5.1	NF1-wild-type tumor cell lines of non-nerve-sheath lineage; in vitro	E3	T3	[75]
Survivin (BIRC5)—3.5.4	MPNST prognostic data from patient specimens; mechanistic link only in RB1-deficient KRAS-mutant lung adenocarcinoma	E3	T3	[76,77,78,79]
Mesenchymal-like (MES-like) versus non-mesenchymal states—3.5.6	NF1-mutant glioblastoma; extrapolated to MPNST	E3	T3	[80,81,82]

## Data Availability

No new data were created or analyzed in this study. Data sharing is not applicable to this article.

## Supplementary Materials

The following supporting information can be downloaded at: https://www.mdpi.com/article/10.3390/cancers18182950/s1, Figure S1: Mechanisms of resistance to MEK inhibitors in NF1-associated plexiform neurofibromas (PN) and malignant peripheral nerve sheath tumours (MPNST). Resistance mechanisms are grouped into six domains corresponding to Section 3.5.1, Section 3.5.2, Section 3.5.3, Section 3.5.4, Section 3.5.5 and Section 3.5.6 of the review: (3.5.1) on-target, pathway-dependent reactivation of MAPK signalling; (3.5.2) parallel, pathway-independent bypass reactivation; (3.5.3) epigenetic and transcriptional rewiring; (3.5.4) cell survival programmes; (3.5.5) tumour microenvironment and immune contexture; and (3.5.6) intratumoral heterogeneity. SC—Schwann cells; TAM—tumour-associated macrophages; ECM—extracellular matrix; RTK—receptor tyrosine kinase; MES—mesenchymal; Table S1: Mechanisms of resistance to MEK inhibitors in plexiform neurofibromas (PN) and MPNST.

## Abbreviations

The following abbreviations are used in this manuscript:

ADCY1	Adenylate cyclase 1
AKT	Protein kinase B
AMPK	AMP-activated protein kinase
ANF	Atypical neurofibroma
ANNUBP	Atypical neurofibromatous neoplasm of uncertain biologic potential
ATG5/ATG7	Autophagy-related proteins 5 and 7
BCRP	Breast cancer resistance protein
BET	Bromodomain and extraterminal domain protein family
BH3	BCL-2 homology domain 3
BIM (BCL2L11)	BCL-2-interacting mediator of cell death
BIRC5	Baculoviral IAP repeat-containing protein 5 (survivin)
BRD2/BRD4	Bromodomain-containing proteins 2 and 4
cAMP	Cyclic adenosine monophosphate
CBP/p300	CREB-binding protein/E1A-binding protein p300
CDK1, CDK4/6	Cyclin-dependent kinases 1, 4 and 6
CDKN2A/B (INK4A/ARF, p16/p14)	Cyclin-dependent kinase inhibitor 2A/B locus
CES	Carboxylesterases
CI	Confidence interval
CRISPR/CRISPRi	Clustered regularly interspaced short palindromic repeats/CRISPR interference
CSF1R	Colony-stimulating factor 1 receptor
CXCL10	C-X-C motif chemokine ligand 10
CYP	Cytochrome P450 enzymes (CYP3A4, CYP2C19)
DUSP	Dual-specificity phosphatase
ECM	Extracellular matrix
EED	Embryonic ectoderm development
eIF4E	Eukaryotic translation initiation factor 4E
ERK	Extracellular signal-regulated kinase 1/2
ESCAT	ESMO Scale for Clinical Actionability of Molecular Targets
EZH1/EZH2	Enhancer of zeste homolog 1/2
FAK	Focal adhesion kinase
FDA	U.S. Food and Drug Administration
FOSL1	FOS-like antigen 1
FOXM1	Forkhead box M1
FOXO3A	Forkhead box O3A
GAP	GTPase-activating protein
GEMM	Genetically engineered mouse model
GRADE	Grading of Recommendations, Assessment, Development and Evaluations
GTP/GDP	Guanosine triphosphate/guanosine diphosphate
H3K27ac	Acetylation of histone H3 at lysine 27
H3K27me3	Trimethylation of histone H3 at lysine 27
HGF	Hepatocyte growth factor
IC_50_	Half-maximal inhibitory concentration
IGF-1R	Insulin-like growth factor 1 receptor
ITGB1	Integrin beta-1
LAMA4	Laminin subunit alpha 4
LKB1	Liver kinase B1
MAPK	Mitogen-activated protein kinase
MCL-1, BCL-2, BCL-xL	Anti-apoptotic proteins of the BCL-2 family
MEK	Mitogen-activated protein kinase kinase 1/2
MEKi	MEK inhibitor
MES-like, NPC-like, OPC-like, AC-like	Mesenchymal-, neural progenitor-, oligodendrocyte progenitor- and astrocyte-like transcriptional cell states
MET	Hepatocyte growth factor receptor
MHC-II	Major histocompatibility complex class II
MIF	Macrophage migration inhibitory factor
MNK1/2	MAPK-interacting kinases 1 and 2
MPNST	Malignant peripheral nerve sheath tumor
mTOR	Mechanistic target of rapamycin
NADPH	Nicotinamide adenine dinucleotide phosphate (reduced)
NCI	National Cancer Institute (USA)
NF1	Neurofibromatosis type 1 (and the gene encoding neurofibromin)
NF2	Neurofibromatosis type 2 (and the gene encoding merlin)
NF-κB	Nuclear factor kappa B
ORR	Objective response rate
PAK	p21-activated kinase
PDGFRβ	Platelet-derived growth factor receptor beta
PD-1/PD-L1	Programmed cell death protein 1 and its ligand
PDOX	Patient-derived orthotopic xenograft
PDX	Patient-derived xenograft
P-gp	P-glycoprotein
PI3K	Phosphoinositide 3-kinase
PKA	Protein kinase A
PN/PNF	Plexiform neurofibroma
PPI	Protein–protein interaction
PPP	Pentose phosphate pathway
PRC2	Polycomb repressive complex 2
PRISMA	Preferred Reporting Items for Systematic Reviews and Meta-Analyses
RAF	Rapidly accelerated fibrosarcoma kinases (ARAF, BRAF, CRAF)
RAP1	Ras-related protein 1
RAS	Rat sarcoma GTPases (KRAS, NRAS, HRAS)
RB1	Retinoblastoma protein 1
RBBP4/7	Retinoblastoma-binding proteins 4 and 7
REiNS	Response Evaluation in Neurofibromatosis and Schwannomatosis
RHO/ROCK	Ras homolog family member/Rho-associated protein kinase
RTK	Receptor tyrosine kinase
RXR	Retinoid X receptor
scRNA-seq/snRNA-seq	Single-cell/single-nucleus RNA sequencing
SHOC2	Soc-2 suppressor of clear homolog
SHP2 (PTPN11)	Src homology 2 domain-containing protein tyrosine phosphatase 2
SOS1	Son of sevenless homolog 1
SPRED	Sprouty-related EVH1 domain-containing protein
SPRY	Sprouty
SRC	Proto-oncogene tyrosine-protein kinase Src
STAT3	Signal transducer and activator of transcription 3
SUZ12	Suppressor of zeste 12
SYRCLE	Systematic Review Centre for Laboratory Animal Experimentation
TAM	Tumor-associated macrophages
TEAD	TEA domain transcription factor
TNBC	Triple-negative breast cancer
TP53/p53	Tumor protein p53
TSC2	Tuberin (tuberous sclerosis complex 2)
UGT	UDP-glucuronosyltransferases (UGT1A6, UGT2B7)
ULK1	Unc-51-like autophagy-activating kinase 1
YAP/TAZ	Yes-associated protein/transcriptional coactivator with PDZ-binding motif
YM155	Sepantronium bromide (survivin suppressant)
