# Supplementary material for: Mechanisms of Resistance to MEK Inhibitors (RAS–MAPK Pathway) in Malignant Peripheral Nerve Sheath Tumors and Their Precursor Lesions (Plexiform Neurofibromas) Associated with Neurofibromatosis Type 1
Source: Cancers (Basel). 2026 Sep 11;18(18):2950. doi: 10.3390/cancers18182950 (PMC13605925; doi:10.3390/cancers18182950)
Supplement: Supplementary file 1 [file cancers-18-02950-s001.zip › MEKi_resistance_mechanisms_mind_map.pdf]

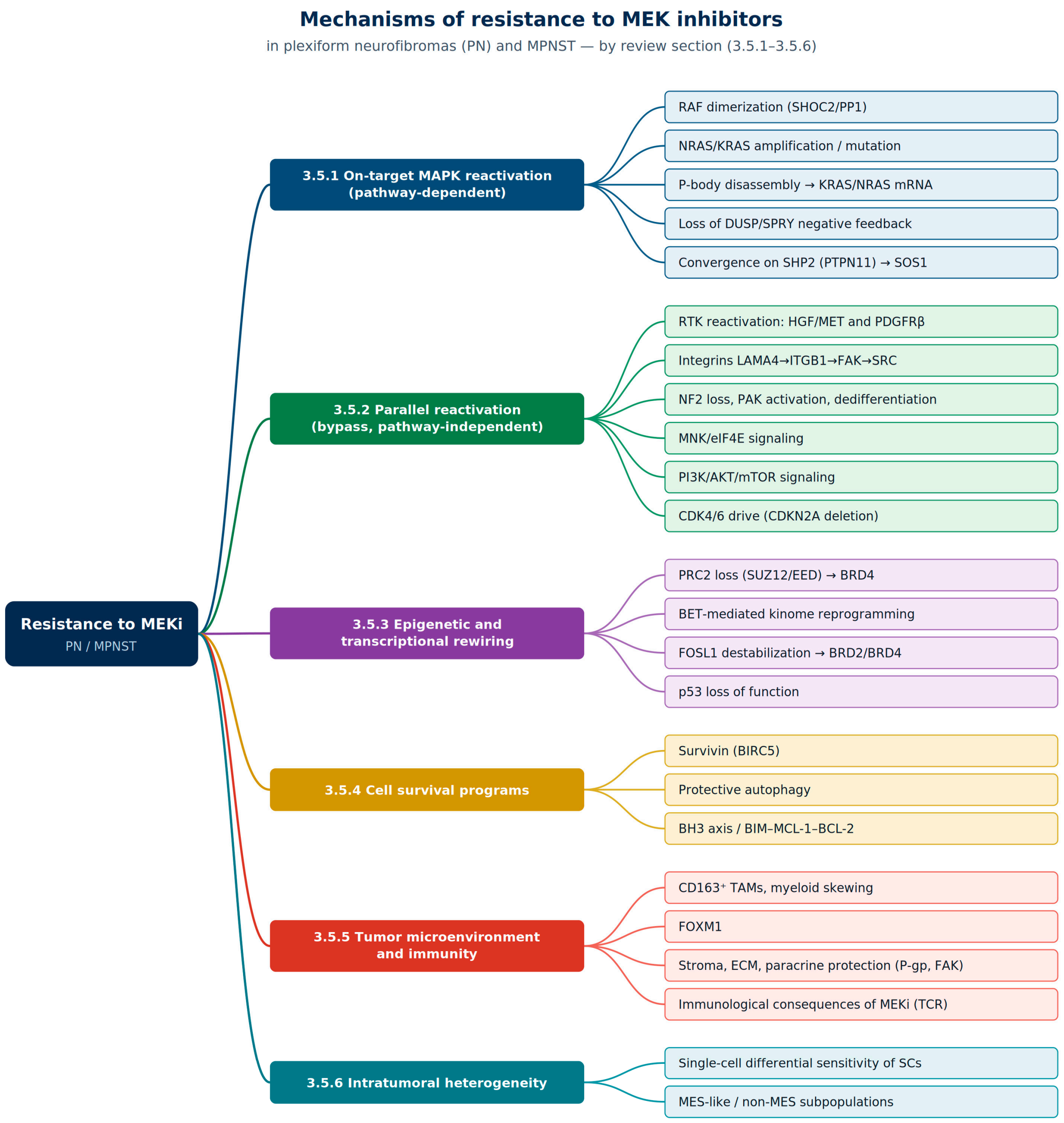

Figure S1. Mechanisms of resistance to MEK inhibitors in NF1-associated plexiform neurofibromas (PN) and malignant peripheral nerve sheath tumours (MPNST). Resistance mechanisms are grouped into six domains corresponding to Sections 3.5.1–3.5.6 of the review: (3.5.1) on-target, pathway-dependent reactivation of MAPK signalling; (3.5.2) parallel, pathway-independent bypass reactivation; (3.5.3) epigenetic and transcriptional rewiring; (3.5.4) cell survival programmes; (3.5.5) tumour microenvironment and immune contexture; and (3.5.6) intratumoral heterogeneity. SC — Schwann cells; TAM — tumour-associated macrophages; ECM — extracellular matrix; RTK — receptor tyrosine kinase; MES — mesenchymal.
