# Supplementary material for: Mechanisms of Resistance to MEK Inhibitors (RAS–MAPK Pathway) in Malignant Peripheral Nerve Sheath Tumors and Their Precursor Lesions (Plexiform Neurofibromas) Associated with Neurofibromatosis Type 1
Source: Cancers (Basel). 2026 Sep 11;18(18):2950. doi: 10.3390/cancers18182950 (PMC13605925; doi:10.3390/cancers18182950)
Supplement: Supplementary file 1 [file cancers-18-02950-s001.zip › MEKi_resistance_table.pdf]

Mechanisms of resistance to MEK inhibitors in plexiform neurofibromas (PN) and MPNST

Summary table of resistance mechanisms grouped by the level at which they operate (Sections 3.5.1–3.5.6). The type of resistance, the model and the method are assigned strictly according to the text of the review.

Resistance mechanism	Type of resistance	Model (PN / MPNST / extrapolation)	Method	References
**3.5.1 On-target MAPK reactivation (pathway-dependent) — the signal is restored within the cascade, bypassing MEK**				
**RAF dimerization (SHOC2/PP1) — including via the SHOC2/PP1 holophosphatase complex**	Adaptive	MPNST (NF1-deficient; mouse models + cell lines)	in vivo / in vitro	[29, 31]
**NRAS/KRAS amplification / mutation (oncogene-level)**	Acquired	Extrapolation: BRAF-mutant melanoma and RAS-mutant colorectal cancer (non-NF1)	in vitro	[30, 32]
**P-body disassembly → KRAS/NRAS mRNA (release of sequestered transcripts)**	Adaptive	Extrapolation: lung adenocarcinoma, melanoma and breast cancer cell lines (non-MPNST)	in vitro	[33]
**Loss of DUSP/SPRY negative feedback (DUSP/SPRY-mediated)**	Adaptive	PN/MPNST (NF1 peripheral nervous system atlas + MPNST cell lines)	in vivo / in vitro	[27, 34, 35]
**Convergence on SHP2 (PTPN11) → SOS1 (reactivation node)**	Adaptive	MPNST (NF1-deficient; mouse models + cell lines)	in vivo / in vitro	[36, 37]
**3.5.2 Parallel reactivation (bypass, pathway-independent) — MAPK re-entry through adjacent inputs: RTKs and parallel effectors**				
**RTK reactivation: HGF/MET and PDGFRβ axes**	Adaptive + acquired	MPNST (NF1-deficient lines selected in vitro; xenografts). Not established as a stand-alone mechanism in PN	in vitro / in vivo	[29, 38, 39, 40]
**Integrins LAMA4→ITGB1→FAK→SRC**	Acquired	MPNST (resistant cell lines)	in vitro	[41]
**NF2 loss, PAK activation, dedifferentiation (of Schwann cells)**	Primary / adaptive	MPNST (dedifferentiation; functional genomic screen + xenografts)	in vivo / in vitro	[31]
**MNK/eIF4E signaling (translational bypass)**	Adaptive	NF1-mutant models (PN/MPNST)	in vivo / in vitro	[42, 43, 44, 45, 46, 47]
**PI3K/AKT/mTOR signaling (parallel survival pathway)**	Primary + adaptive	MPNST (cell lines) + negative clinical trial (selumetinib + sirolimus)	in vitro + clinical trial	[48, 49, 50]
**CDK4/6 drive (CDKN2A deletion) — CDK-dependent mechanisms**	Primary	MPNST (predominantly; the locus is retained in intact PN)	in vitro + in vivo	[51]
**3.5.3 Epigenetic and transcriptional rewiring — resistance at the level of gene expression control**				
**PRC2 loss (SUZ12/EED) → BRD4, via increased H3K27ac**	Primary	MPNST (mouse models)	in vivo	[52, 53]
**BET-mediated kinome reprogramming (RTK induction)**	Adaptive	Extrapolation: TNBC (cell lines + GEMM); direct transfer to PN/MPNST not shown	in vitro + in vivo	[55]
**FOSL1 destabilization → BRD2/BRD4-mediated RTK induction**	Primary	Extrapolation: NF1-deficient ovarian cancer; transfer to MPNST not proven	in vitro	[56]
**p53 loss of function (signal shift from RAS/ERK to PI3K/AKT)**	Primary	MPNST (NF1-associated mouse models + isogenic CRISPR p53 knockout + human cell lines)	in vitro / in vivo	[49]
**3.5.4 Cell survival programs — evasion of cell death, apoptotic blockade, catabolic survival**				

Resistance mechanism	Type of resistance	Model (PN / MPNST / extrapolation)	Method	References
**Survivin (BIRC5) — anti-apoptotic programs (candidate mechanism)**	Primary (candidate)	MPNST (prognostic data, patient samples); mechanistic basis extrapolated from Sumi et al. in non-MPNST cells	in vitro / patient samples	[57, 58, 59, 60]
**Protective autophagy (cytoprotective)**	Adaptive / acquired (not established for MEKi in MPNST)	Extrapolation: melanoma/pancreatic/colorectal PDX + BRAF-mutant melanoma (BAMM) paradigm; in MPNST shown only for SHP2i	in vitro / in vivo + clinical (BAMM)	[39, 61, 62, 63]
**BH3 axis / BIM–MCL-1–BCL-2 (BIM sequestration, elevated BCL-xL)**	Adaptive / primary	MPNST (direct expression data, NF1 deficiency, in vitro / patient samples); BH3 mechanics extrapolated	in vitro / patient samples	[64, 65, 66, 67]
**3.5.5 Tumor microenvironment and immunity — resistance residing outside the tumor cell**				
**CD163^{+} TAMs, myeloid skewing — myeloid immune evasion**	Primary	MPNST (immunophenotyping of surgical specimens); PN (mouse models)	clinical (specimens) / in vivo	[37, 68, 69, 70]
**FOXM1 as a mediator of escape and immune evasion**	Acquired	MPNST (preclinical); the PD-L1 link is partly extrapolated; demonstrated under CDK4/6 + MEK, not under MEKi monotherapy	in vitro / in vivo	[51, 71]
**Stroma, ECM, paracrine protection (P-gp, FAK) — paracrine shielding from MEKi via P-gp efflux and FAK signaling**	Primary	PN (3D co-cultures with patient-derived fibroblasts; hydrogels of defined stiffness)	in vitro (3D)	[72, 73, 74]
**Immunological consequences of MEKi (TCR) — suppression of MEK/ERK signaling downstream of the TCR (pro-resistance component)**	Adaptive	Extrapolation: mechanism defined outside NF1 (melanoma/lung/pancreas); in NF1 only within combination regimens	in vivo / extrapolation	[75, 76, 77, 78, 51]
**3.5.6 Intratumoral heterogeneity — a source of incomplete response to MEKi**				
**Single-cell differential sensitivity of SCs (Schwann cells)**	Primary (pre-existing heterogeneity)	Predominantly PN (not studied in MPNST); transcriptional surrogates + xenografts	in silico (scRNA-seq/snRNA-seq) / in vivo	[31, 79]
**MES-like / non-MES subpopulations**	Primary, with adaptive RAS reactivation	Extrapolation: NF1-mutant glioblastoma; transfer to PN/MPNST not proven (MEKi response untested in MPNST, no data for PN)	snRNA-seq/scRNA-seq + CRISPRi; in vivo (mouse models)	[80, 81, 82]

Note: "Extrapolation" indicates that the mechanism has been characterized in a different tumor entity or model system and has not been confirmed directly in PN/MPNST (the primary source is specified in the "Model" column). Reference numbering follows the reference list of the main document.
